# The effectiveness of biophysical agents in the treatment of carpal tunnel syndrome- an umbrella review

**DOI:** 10.1186/s12891-023-06778-z

**Published:** 2023-08-10

**Authors:** Armaghan Dabbagh, Christina Ziebart, Joy C MacDermid, Tara Packham, Ruby Grewal

**Affiliations:** 1 Department of Rehabilitation Sciences, Faculty of Health Sciences, Elborn College, Western University, London, ON Canada; 2 KITE-UHN, University of Toronto, Toronto, ON Canada; 3 Department of Physical Therapy, Faculty of Health Sciences, Western University, London, ON Canada; 4 Roth McFarlane Hand and Upper Limb Centre, St. Joseph’s Hospital, London, ON Canada; 5 School of Rehabilitation Science, McMaster University, Hamilton, ON Canada; 6 Department of Surgery, Western University, London, ON Canada

**Keywords:** Carpal tunnel syndrome, Biophysical agents, Low-level laser therapy, Ultrasound, Diathermy

## Abstract

**Background:**

Our objective was to summarize, synthesize, and integrate the evidence evaluating the effectiveness of biophysical agents compared to other conservative treatments, for the management of carpal tunnel syndrome (CTS).

**Methods:**

This was an overview of systematic reviews (SRs). We searched several online databases and obtained SRs relating to managing CTS using biophysical agents. Two independent researchers screened and appraised the quality of the SRs using the A MeaSurement Tool to Assess systematic Reviews-2 appraisal tool. We extracted information related to study characteristics as well as the effectiveness of biophysical agents for CTS, the effect sizes, and between-group significances. We categorized the information based on the type of biophysical agent. We also performed a citation mapping and calculated the corrected covered area index.

**Results:**

We found 17 SRs addressing 12 different biophysical agents. The quality of the SRs was mainly critically low (n = 16) or low (n = 1). The evidence was inconclusive for the effectiveness of Low-level Laser therapy and favorable for the short-term efficacy of non-thermal ultrasound in improving symptom severity, function, pain, global rating of improvement, satisfaction with treatment, and other electrophysiological measures compared to manual therapy or placebo. Evidence was inconclusive for Extracorporeal Shockwave therapy, and favorable for the short-term effectiveness of Shortwave and Microwave Diathermy on pain and hand function. The corrected covered area index was lower than 35% indicating a low overlap of the SRs.

**Conclusions:**

The findings were based on low-quality primary studies, with an unclear or high risk of bias, small sample sizes, and short follow-ups. Therefore, no recommendations can be made for the long-term effectiveness of any biophysical agents. High-quality evidence is needed to support evidence-based recommendations on the use of biophysical agents in the management of CTS.

**PROSPERO registration number:**

CRD42022319002, registered on 17/04/2022

**Supplementary Information:**

The online version contains supplementary material available at 10.1186/s12891-023-06778-z.

## Introduction

Carpal tunnel syndrome (CTS) accounts for 90% of all upper extremity neuropathies [1]. Compression or traction of the median nerve as it passes from the forearm towards the hand, underneath the transverse carpal ligament is implicated as a causal mechanism [2, 3]. The pathogenesis of CTS also includes unbalanced tension of the epimysial fasciae that limits nerve displacement in CTS cases [4]. CTS is one of the most common disabling upper extremity conditions among workers, and accounts for a large portion of worker compensations claims [5–7]. The symptoms include tingling and numbness, in digits innervated by the median nerve [8–10]. Moreover, fine manual dexterity can be impaired in CTS cases, that affects the performance in daily living activities, hobbies, and work, especially in activities that require dexterity such as writing and handling small objects such as coins, cups, or tools [11].

According to Baker et al. 2011, “CTS is a complex condition with a wide variety of treatments provided by a multitude of disciplines.” [12] The diagnostic options range from diagnostic questionnaires and physical examinations to more invasive methods such as nerve conduction velocity testing [8, 13, 14]. When diagnosed early, conservative treatments are usually the first line of management. However, with more severe cases, carpal tunnel release surgery might be inevitable [15]. Several different conservative treatment options have been summarized in the 2019 clinical practice guidelines of the American Physical Therapy Association [16]. These treatment options include manual therapy, exercise, education and ergonomic evaluation, and biophysical agents, etc. [16, 17] Other more recent treatment methods include the injection of Botulinum Toxin, Corticosteroids, and Acupuncture [17–20].

Biophysical agents are one of the most routinely used management techniques in physiotherapy, occupational therapy and hand therapy practice settings for people with CTS [21]. According to the American Physical Therapy Association, these techniques include electrophysical modalities such as interferential currents, and transcutaneous electrical nerve stimulation (TENS); sound agents (ultrasound); light agents such as low-level laser therapy (LLLT), and non-laser light therapy; thermal agents such as contrast baths and heat wrap therapy; and athermal agents such as magnet therapy; and transdermal drug delivery [16, 21–23].

The effectiveness of biophysical agents for the treatment of CTS has been evaluated in multiple systematic reviews (SRs) with varying qualities and performance across studies [23–29]. Umbrella reviews are a form of synthesis that are used to derive recommendations from the larger pool of evidence within the reviews, acknowledging that some reviews will contain overlapping primary evidence, and some unique aspects of studies are included on how the evidence is evaluated or synthesized. The primary objective of this study was to provide a comprehensive and systematic integration of the evidence regarding non-surgical biophysical interventions of CTS, from published SRs, through conducting an umbrella review. The secondary objective was to analyze and compare findings from different SRs addressing the same biophysical interventions to assist clinicians with evidence-based decision-making in their clinical practice.

## Methods

This is an umbrella review: an overview of SRs. We registered the protocol for this review with PROSPERO (CRD42022319002) on 17/04/2022.

### Information sources

We comprehensively searched relevant SRs in CINAHL, Medline, and EMBASE through Ovid and the Cochrane database of systematic reviews from inception. We also searched the PROSPERO registry of systematic reviews and did a hand search of the final included articles. We developed our search strategy in consultation with a health sciences librarian at Western University and conducted our electronic database search on November 19, 2021. The search was updated on February 22, 2023. We created three search clusters combining MESH terms and keywords relating to CTS treatment and used OR function within the clusters, then AND function between the clusters to combine them. The three search clusters were related to (1) CTS, (2) treatments, and (3) SRs (APPENDIX I). To limit our search results to only SRs, we adopted some keywords from the CADTH strings attached search terms for SRs [30].

### Study selection

Two authors (AD, CZ) independently selected the studies in two consecutive phases. In the first phase, we screened the titles and abstracts. In this phase, we removed the studies whose titles and abstracts did not meet the eligibility criteria. In the second phase, we retrieved the full texts of the remaining articles and reviewed them against the eligibility criteria. In each of these phases, if a disagreement occurred, we consulted the senior co-author (JM) and resolved the dispute through discussion, however there were no articles that resulted in a disagreement.

### Eligibility criteria

We included all SRs that fulfilled the following inclusion criteria.

**Design**: systematic reviews, with or without metanalysis that included primary papers of experimental study designs.

**Population**: SRs that included people with CTS. In cases where SRs addressed broader populations such as upper limb neuropathies or MSK disorders, we included and reported the data for the CTS subpopulation.

**Intervention**: eligible SRs addressed non-surgical interventions as a sole treatment or combinations of different non-surgical interventions for CTS. It included the non-surgical biophysical agent interventions as summarized by the American Physical Therapy Association clinical practice guidelines: [16]

- Thermotherapy: dry heat, paraffin, microwave, and shortwave diathermy (MWD, SWD), heat wrap therapy, contrast bath.
- Electrical stimulations: interferential currents and TENS.
- Light agents: LLLT and non-laser light therapy.
- Sound agents: ultrasound.
- Transdermal drug delivery: topical anti-inflammatory drugs, Phonophoresis, Iontophoresis.
- Athermal agents: magnet therapy, pulsed radiofrequency.

In addition to the above-mentioned biophysical agents, we also included Extracorporeal shockwave therapy (ESWT), even though this was not addressed as a physiotherapy modality in the American Physical Therapy Association clinical practice guidelines.

**Comparison**: all surgical and non-surgical interventions (manual therapy, local steroid injections, etc.) for managing CTS were considered eligible comparators.

**Outcome**: all outcomes addressing the short- and long-term effectiveness and potential adverse effects of non-surgical interventions were eligible. These include patient-centered (e.g., quality of life, pain, function) and secondary, surrogate, or intermediate outcomes (e.g., electromyography, nerve conduction velocity testing). As a criterion of failure of non-surgical interventions, the number of surgeries or the need for surgery (number of treatment sessions needed to avoid one surgery) was considered when reported.

**Time**: any time frame. If the authors updated the systematic reviews, we only kept the most recent version.

**Exclusion criteria**: no exclusions based on sample size, age and gender of the participants, the severity of CTS, and the time of publication were made. We excluded gray literature, conference presentations (e.g., abstracts, posters), unpublished manuscripts, dissertations, books and book chapters, meeting abstracts, and consensus development statements. Further, we excluded cadaveric or animal studies, diagnostics, prognosis, screening, economic analysis, or any intervention other than biophysical agents for CTS (e.g., manual therapy, exercise, education, splint, etc.).

### Data extraction

We used a pre-developed data extraction sheet and registered it on the PROSPERO. One author (CZ) extracted the data from all included SRs. Another author (AD) did a duplicate extraction and verified all the extracted data. We extracted data from the included SRs and not from the primary studies within SRs, as per the 2021 guidelines by Cochrane for overviews of reviews [31]. The extracted data included information relating to the SRs (authors, year, count and type of the primary studies, etc.), patients (age, CTS severity, sex, or gender, etc.), and biophysical agents (type, effectiveness, comparison, etc.).

### Data synthesis and analysis

We categorized the extracted information according to the different types of biophysical agents [16], and reported them in the results section in order of frequency. For SRs performing meta-analysis, we extracted and reported the effect sizes, and between group significances based on the outcome measure that was used in the SR. We examined the overlap of the primary studies by creating a citation matrix of the primary studies. We followed Hennessy and Johnson 2019 recommendations for calculating a corrected covered area (CCA) index [32]. This approach is recommended when there are several SRs on the same topic, and the primary studies might overlap [32]. We used the following formula to calculate the CCA index:

$$CCA\, = \,\frac{{{\rm{Total}}\,{\rm{n}}\,{\rm{of}}\,{\rm{included}}\,{\rm{primary}}\,{\rm{studies}}\, - \,{\rm{n}}\,{\rm{of}}\,{\rm{rows}}}}{{\left( {n\,of\,rows\, \times \,n\,of\,columns} \right)\, - \,n\,of\,rows}}$$

In this formula, total number of included primary studies included the double counting, the number of rows refers to the primary studies, and number of columns is the number of SRs [32]. We calculated the CCA index when three or more SRs addressed the same intervention.

### Quality assessment

Two co-authors (CZ, AD) independently critically appraised the quality of the included SRs, using the “A MeaSurement Tool to Assess systematic Reviews-2” (AMSTAR-2) appraisal tool [33]. AMSTAR-2 tool has 16 items, which were rated as “yes” (denotes positive results), “no” (denotes negative results), and not applicable [33].

Seven of the 16 items of the AMSTAR-2 are considered as critical domains, which are items 2, 4, 7, 9, 11, 13, 15 [33]. Overall, if a SR was rated yes in one of these critical items, it was regarded as having ‘low’ overall confidence in the results. If a SR had more than one critical flaw, it was rated as ‘critically low’. On the other hand, if a SR did not have any critical or only one non-critical flaws or if a SR only more than one non-critical flaws, it was regarded as having ‘high’ or ‘moderate’ overall confidence in the results of the review [33].

## Results

### Study selection

**Fig. 1:**
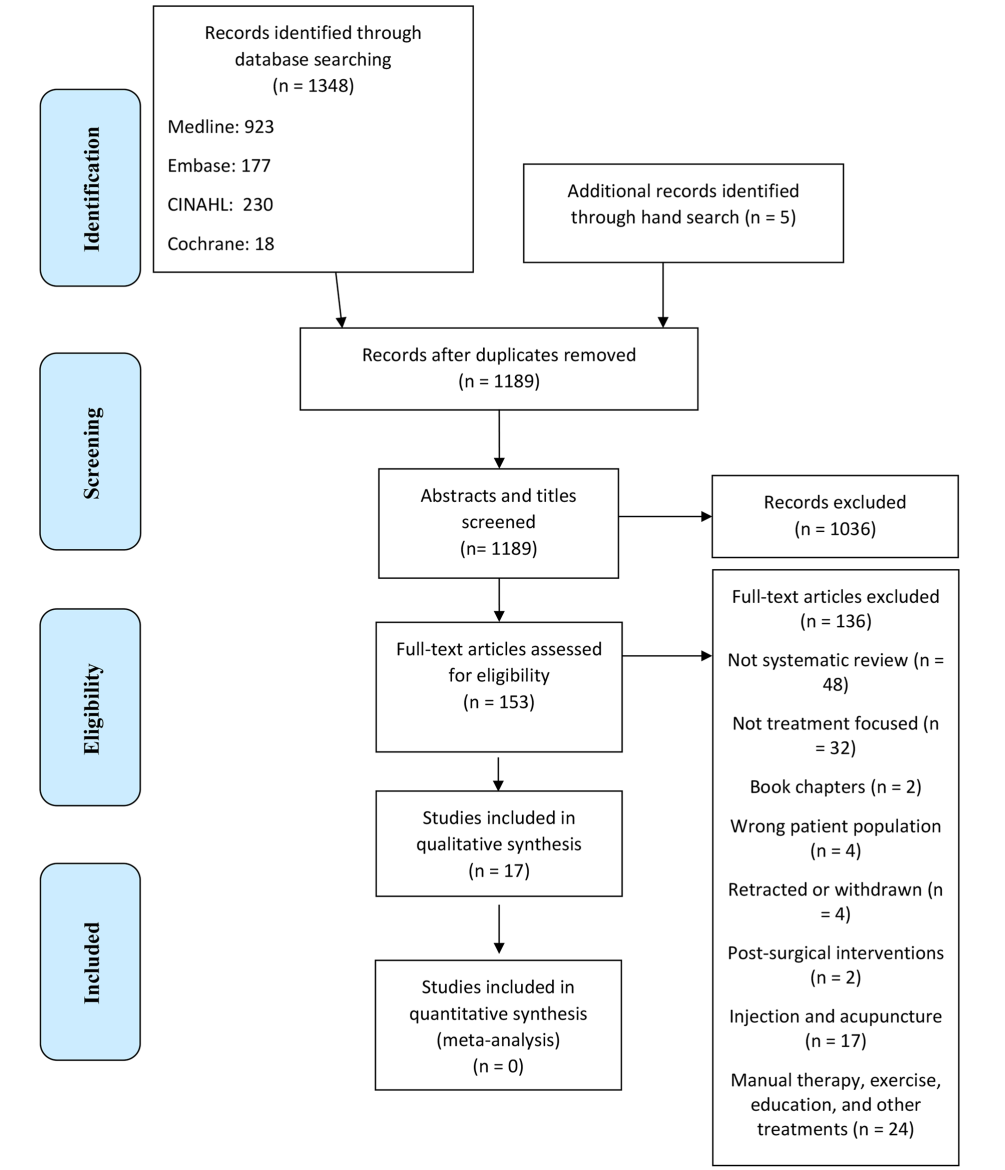
PRISMA diagram

We obtained 1348 citations through the electronic database search. After removing the duplicates, we screened 1189 articles in the first phase. We then proceeded to the full-text reviewing phase with 153 full-text articles. Lastly, 17 SRs met all the eligibility criteria for our overview. Exclusion reasons and a full list of excluded articles (title, authors, doi) after full-text review are presented in Appendix II. The Kappa agreement between the reviewers in the first phase was 0.82 (SE: 0.03, 95% CI 0.75–0.87), which indicates strong agreement. Please refer to Fig. 1, the PRISMA diagram, to see the detailed study selection process.

### Study characteristics

Among the 17 included SRs, 10 conducted a meta-analysis [27–29, 34–40]. Only five SRs had registered their protocols, five in PROSPERO [27, 28, 34, 39, 40], and one in INPLASY [37]. All of the SRs had searched at least four online databases, and the database in common was Medline/PubMed. After removing the duplicates, an overall of 68 primary original studies were included in the reviews which are summarized in Appendix III in alphabetical order for each treatment modality. The population under study was people with CTS in 11 SRs [23–25, 27–29, 36–40], any population with pain or MSK disorders in three SRs [35, 41, 42], peripheral somatosensory neuropathy or injury in two SRs [26, 43], and radial, ulnar, and median neuropathies in one SR [34]. The study characteristics are summarized in in Table 1.

**Table 1:** Characteristics of Included Systematic Reviews

First author and year	Design	Protocol registered	Date of search	Databases searched	No. of primary studies	No. of Participants/ age/ sex or gender	Population	Intervention/s	ROB tool (evidence rating)	AMSTAR-2 rating
Bekhet 2017 [27]	SR & MA	PROSPERO (CRD42016050283)	Apr 2016	PubMed, Web of Knowledge, Scopus, Cochrane Central, and VHL	8 RCTs	473 patients/ 631 wrists/ age range of 35 to 64	CTS	LLLT	Cochrane 7-item ROB criteria (unclear ROB)	Critically low quality
Bula-Oyola 2021 [34]	SR & MA	PROSPERO (CRD42020168792)	Apr & Jul 2019	Biomed Central, Ebscohost, Lilacs, Ovid, PEDro, Sage, Scopus, Science Direct, Semantic Scholar, Taylor & Francis, Web of Science	38 RCTs in total, 34 RCTs in CTS	1766 participants	Radial, ulnar, and median neuropathies	LLLT, ESWT, US, static and pulsed magnetic fields, PPNL, SWD	GRADE (low or very low quality)	Critically low quality
Burger 2017 [25]	SR	No	Mar 2015	CINAHL, Cochrane Library, EBSCOhost, PEDro, PubMed, Science Direct, Scopus	9 RCTs	614 participants/ age range of 43- 52.6	CTS	LLLT	PEDro scale (8.2/10, low quality)	Critically low quality
Cheung 2020 [28]	SR & MA	PROSPERO (CRD42017082650)	NR	Cochrane Central Register of Controlled Trials, MEDLINE, EMBASE, PsycINFO	6 RCTs	418 patients	CTS	LLLT	Cochrane 7-item ROB criteria (moderate ROB)	Critically low quality
Fallah 2017 [43]	SR	No	Oct 2015	PubMed (Medline), Cochrane library, PT Evidence Database	10 RCTs, 6 in CTS	229 hands in CTS	Peripheral somatosensory neuropathy	LLLT	NR	Critically low quality
Franke 2018 [24]	SR	No	Apr 2016	The Cochrane Library, PubMed, Embase, CINAHL, PT Evidence Database	17 RCTs	984 participants	CTS	LLLT	Cochrane 2009 criteria (strong evidence)	Critically low quality
Fu 2019 [26]	SR	No	Apr 2019	EMBASE, MEDLINE, BIOSIS Previews, PubMed, Web of Science	11 in total, 4 RCTs in CTS	128 participants, 207 wrists with CTS	Peripheral nerve injury	SWD, MWD	NR	Critically low quality
Fulop 2010 [35]	SR & MA	No	NR	Medline, PubMed, Ovid, PsycInfo	22 RCTs, 1 RCT in CTS	19 participants with CTS	Any population with pain	LLLT	NR	Critically low quality
Huisstede 2018 [23]	SR	No	Apr 2016	Cochrane Library, PubMed, Embase, CINAHL, PT Evidence Database	22 RCTs	1652 participants	CTS	US, ESWT, heat wrap therapy, local microwave hyperthermia, iontophoresis, PRF, SWD, IFC, TENS, magnets	Cochrane 2009 criteria (moderate evidence)	Critically low quality
Kim 2019 [36]	SR & MA	No	Aug 2018	PubMed-Medline, Embase, Cochrane Library	6 RCTs	281 participants	CTS	ESWT	Cochrane 7-item ROB criteria (low ROB)	Critically low quality
Li 2020 [37]	SR & MA	INPLASY 202,080,025	Sept 2020	PubMed, Embase, Cochrane Library, China National Knowledge Infrastructure database (CNKI), WanFang database, Chinese Scientific Journal Database	5 RCTs	204 participants	CTS	ESWT	Cochrane 7-item ROB criteria (low and unclear ROB)	Low quality
Li 2016 [38]	SR & MA	No	NR	PubMed, Medline, EMBASE, Science Direct	7 RCTs	491 wrists	CTS	LLLT	Cochrane 7-item ROB criteria (low and unclear ROB)	Critically low quality
Page 2013 [29]	SR & MA	No	Nov 2012	Cochrane Neuromuscular Disease Group Specialized Register, CENTRAL, MEDLINE, EMBASE, CINAHL, AMED	11 RCTs	414 participants	CTS	US	Cochrane 7-item ROB criteria (unclear and high ROB)	Low quality
Rankin 2017 [39]	SR & MA	PROSPERO (CRD42016037433)	Dec 2016	CENTRAL, MEDLINE, Embase, Science Citation Index Expanded for RCTs	22 RCTs	1153 participants	CTS	LLLT	Cochrane 7-item ROB criteria (unclear and high ROB)	Critically low quality
Robertson 2001 [41]	SR	No	NR	MEDLINE, CINHAL	10 in total, 1 RCT on CTS	NR	Patients with pain or a MSK disorder	US	NR	Critically low quality
Roll 2017 [42]	SR	No	NR	MEDLINE, PsycINFO, CINAHL, Ergonomics Abstracts, OTseeker	59 in total, 10 RCTs, and 1 cohort studies on CTS	NR	Adults with MSK disorders of the forearm, wrist, and hand	LLLT, US, heat wrap, phonophoresis, iontophoresis	Cochrane 7-item ROB criteria (varied ROB)	Critically low quality
Xie 2023 [40]	SR & MA	PROSPERO (CRD42019119841)	Dec 2019	Medline, Embase, PEDro, CENTRAL, OpenGrey, CNKI, VIP, Wang Fang databases, and China Biological Medicine	10 RCTs	433 patients (501 wrists), mean age ranging from 46 to 60 years old	CTS	ESWT	Cochrane 7-item ROB criteria (low and moderate ROB), PEDro (scores ranged from 4 to 9)	Critically low quality

*List of abbreviations*: AMED, Allied and Complementary Medicine Database; CINAHL, Cumulative Index to Nursing and Allied Health Literature; CTS, carpal tunnel syndrome; ROB, risk of bias; VHL, virtual health library; ESWT, extracorporeal shockwave therapy; IFC, interferential currents; LLLT, low-level laser therapy; MA, meta-analysis; MEDLINE, Medical Literature Analysis and Retrieval System Online; MWD, microwave diathermy; MSK, musculoskeletal;NR, not reported; OT, occupational therapy; PEDro, Physiotherapy Evidence Database; PT, physical therapy; PPNL, polarized polychromatic noncoherent light (bioptron) therapy; PRF, pulsed radiofrequency; ROB, risk of bias; SciELO, Scientific Electronic Library Online; SR, systematic review; SWD, shortwave diathermy; TENS, transcutaneous electrical stimulation; US, ultrasound; WoS, Web of Science.

### Overall confidence in the results of the systematic reviews (AMSTAR-2)

Of the 17 reviews, none was classified as having high or moderate quality. The quality of the SRs was low in one article [29, 37], and critically low in the remaining 16 studies. Most studies had not established or registered a protocol before conducting their review, therefore, it was not possible to track or justify deviations from the protocol. This introduces a risk of selective reporting by the SR authors. We rated studies as ‘no’ in item 7 because the authors did not provide enough details regarding the included studies. Most studies did not provide a list of excluded articles and the exclusion reasons. Lastly, regarding item 13, we rated 12 items as ‘no’ because the authors did not recognize or discuss the impact of the ROB of the primary studies in their results and conclusion. The full AMSTAR-2 rating report is presented in Table 2.

**Table 2:** AMSTAR-2 Ratings of the Included Systematic Reviews

Studies	1	2	3	4	5	6	7	8	9	10	11	12	13	14	15	16	Overall quality
Bekhet 2017 [27]	Y	Y	N	PY	Y	Y	N	Y	Y	N	Y	Y	N	Y	N	Y	Critically low
Bula-Oyola 2021 [34]	Y	Y	N	PY	Y	Y	N	N	Y	N	Y	N	N	Y	N	Y	Critically low
Burger 2017 [25]	Y	N	N	PY	Y	Y	N	Y	Y	N	N/A	N/A	N	Y	N/A	Y	Critically low
Cheung 2020 [28]	Y	Y	N	PY	Y	Y	Y	Y	Y	N	Y	N	N	Y	N	Y	Critically low
Fallah 2017 [43]	Y	N	N	PY	Y	Y	N	Y	N	N	N/A	N/A	N	Y	N/A	Y	Critically low
Franke 2018 [24]	Y	N	Y	PY	Y	Y	N	Y	Y	N	N/A	N/A	N	Y	N/A	N	Critically low
Fu 2019 [26]	Y	N	N	PY	N	N	N	PY	N	Y	N/A	N/A	N	Y	N/A	Y	Critically low
Fulop 2010 [35]	Y	N	Y	PY	N	N	N	PY	N	N	Y	N	N	Y	Y	N	Critically low
Huisstede 2018 [23]	Y	N	N	PY	Y	Y	PY	PY	Y	N	N/A	N/A	Y	Y	N/A	Y	Critically low
Kim 2019 [36]	Y	N	Y	Y	Y	Y	Y	Y	Y	Y	Y	N	N	Y	N	Y	Critically low
Li 2020 [37]	Y	Y	Y	Y	Y	Y	N	Y	Y	Y	Y	Y	N	Y	Y	Y	Critically Low
Li 2016 [38]	Y	N	Y	PY	Y	Y	N	Y	Y	Y	Y	N	Y	Y	N	Y	Critically low
Page 2013 [29]	Y	N	Y	PY	Y	Y	Y	Y	Y	N	Y	Y	Y	Y	Y	Y	Low
Rankin 2017 [39]	Y	N	Y	Y	Y	Y	Y	Y	Y	Y	Y	Y	Y	Y	N	Y	Critically low
Robertson 2001 [41]	Y	N	Y	N	Y	Y	N	N	N	N	N/A	N/A	N	N	N/A	N	Critically low
Roll 2017 [42]	Y	N	Y	Y	Y	N	N	Y	Y	N	N/A	N/A	N	Y	N/A	Y	Critically low
Xie 2022 [40]	Y	Y	Y	PY	Y	Y	N	Y	Y	N	Y	N	N	Y	N	Y	Critically low

*Abbreviations*: Y, yes; N, no; PY, partially yes; N/A, not applicable.

Rating items: “1. Did the research questions and inclusion criteria for the review include the components of PICO? 2. Did the report of the review contain an explicit statement that the review methods were established prior to the conduct of the review and did the report justify any significant deviations from the protocol? 3. Did the review authors explain their selection of the study designs for inclusion in the review? 4. Did the review authors use a comprehensive literature search strategy? 5. Did the review authors perform study selection in duplicate? 6. Did the review authors perform data extraction in duplicate? 7. Did the review authors provide a list of excluded studies and justify the exclusions? 8. Did the review authors describe the included studies in adequate detail? 9. Did the review authors use a satisfactory technique for assessing the risk of bias (RoB) in individual studies that were included in the review? 10. Did the review authors report on the sources of funding for the studies included in the review? 11. If meta-analysis was performed, did the review authors use appropriate methods for statistical combination of results? 12. If meta-analysis was performed, did the review authors assess the potential impact of RoB in individual studies on the results of the meta-analysis or other evidence synthesis? 13. Did the review authors account for RoB in primary studies when interpreting/discussing the results of the review? 14. Did the review authors provide a satisfactory explanation for, and discussion of, any heterogeneity observed in the results of the review? 15. If they performed quantitative synthesis did the review authors carry out an adequate investigation of publication bias (small study bias) and discuss its likely impact on the results of the review? 16. Did the review authors report any potential sources of conflict of interest, including any funding they received for conducting the review?

### Risk of bias and quality assessment tools in the included systematic reviews

Thirteen SRs used five different ROB or quality assessment tools, as summarized below in order of frequency. Four SRs did not report or perform quality or ROB appraisals [26, 35, 41, 43].

#### Cochrane 7-item criteria

Nine SRs used the Cochrane 7-item ROB assessment criteria [27–29, 36–40, 42]. All nine articles cited the ROB assessment criteria published in 2008 by Higgins and Altman [44]. The assessment criteria in this appraisal tool are “sequence generation, allocation sequence concealment, blinding of participants, personnel and outcome assessors, incomplete outcome data, selective outcome reporting, and other potential threats to validity” [44].

#### Cochrane 2009 criteria

Two studies used a modified version of the 2009 Cochrane criteria to assess the overall quality of the evidence [45]. Both studies adapted the seven items proposed by Furlan et al. and added five extra items [23, 24]. The twelve assessment items were “adequate randomization, allocation concealment, blinding patients, blinding caregivers, blinding outcome assessors, incomplete outcome data addressed (dropouts), incomplete outcome data (ITT analysis), free of suggestions of selective outcome reporting, similarity of baseline characteristics, cointerventions avoided or similar, compliance acceptable in all groups, timing of the outcome assessment similar” [24]. Both studies set a threshold of 50% to define high quality evidence [23, 24].

#### Grading of recommendations assessment, development and evaluation

Only Bula-Oyola et al.’s study used the GRADE tool to summarize the quality of the evidence [46]. They used GRADEpro GDT software (gradepro.org/) to assess the quality and generate the summary tables [34]. GRADE tool assesses the quality of evidence based on the following criteria: “risk of bias, inconsistency, indirect evidence, imprecision, and other considerations (including publication bias, large effect, plausible confounding, and dose-response gradient).” [34]

#### PEDro scale

Two studies [40, 47] used the PEDro scale to rate the methodological quality and risk of bias of the included primary studies [48]. The PEDro scale is a 11-item scale, appraising the internal validity, statistical reporting, and external validity.

### Biophysical agents

In the following sections, a narrative summary of all the included biophysical agents is provided in order of the frequency. More detailed information can be found in Table 3.

**Table 3:** Effects of biophysical agents on CTS Management

Study	Treatment vs. control (follow-up period)	No. of studies (participants)	Outcome (or outcome measure)	Effect size (mean difference, 95% confidence intervals)	Significant between group difference	Result or conclusion
Bekhet 2017 [27]	LLLT vs. control	4 (NR)	Pain (VAS)	−1.11 (− 2.60, 0.37), p = 0.14	No	“Our results showed that LLLT was superior to placebo in terms of improving the grip strength in patients with mild to moderate CTS. However, both groups were comparable in terms of pain reduction, functional status improvement, and other electrophysiological measures (sensory and motor distal latencies, and CMAP) after follow-up for 3 months” pg. 1444
	LLLT vs. control	5 (NR)	Function (FSS)	−1.33 (− 3.30, 0.65), p = 0.18	No	
	LLLT vs. control	5 (NR)	Symptom severity (SSS)	−1.42 (− 5.17, 2.33), p = 0.45	No	
	LLLT vs. control	6 (NR)	Grip strength	19.20 (1.63, 2.75), p < 0.001	Favours LLLT	
	LLLT vs. control	3 (NR)	SNAP	-2.71 (− 3.62, − 1.80), p < 0.001	No	
	LLLT vs. control	4 (NR)	CMAP	0.03 (− 0.16, 0.22), p = 0.77	No	
	LLLT vs. control	4 (NR)	Sensory distal latency	-0.02 (− 0.20, 0.15), p = 0.07	No	
	LLLT vs. control	8 (NR)	Motor distal latency	-0.31 (− 0.77, 0.15), p = 0.21	No	
Bula-Oyola 2021 [34]	Electrophysical modalities vs. placebo	17 (700)	Pain (VAS)	-0.89 (-1.79, 0.02)	Favors EM	“Low-level laser therapy and ultrasound showed favourable results in improving symptom severity and functional status compared to manual therapy. In addition, the low-level laser showed improvements in pinch strength compared to placebo and pain (VAS) compared to manual therapy. Splints showed superior results to electrophysical modalities. The clinical significance of the results was assessed by effect size estimation and comparison with the minimum clinically important difference” pg. 1.
	Electrophysical modalities vs. placebo	17 (747)	Symptom severity (SSS)	-1.01 (-1.65, -0.37)	Favors EM	
	Electrophysical modalities vs. placebo	15 (639)	Functional status (FSS)	-0.79 (-1.45, -0.13)	Favors EM	
	Electrophysical modalities vs. placebo	15 (713)	Sensory latency	0.03 (-0.29, 0.35)	Favours placebo	
	Electrophysical modalities vs. placebo	20 (912)	Motor latency	-0.3 (-0.66, 0.04)	No	
	Electrophysical modalities vs. placebo	17 (719)	Sensory velocity	0.09 (-0.57, 0.38)	No	
	Electrophysical modalities vs. placebo	9 (354)	Motor velocity	0.27 (-0.26, 0.80)	No	
	Electrophysical modalities vs. placebo	5 (333)	SNAP amplitude	0.28 (-0.06, 0.62)	No	
	Electrophysical modalities vs. placebo	6 (371)	CMAP amplitude	0.15 (-0.41, 0.72)	No	
	Electrophysical modalities vs. placebo	6 (383)	Grip strength	0.08 (-0.27, 0.42)	No	
	Electrophysical modalities vs. placebo	3 (227)	Pinch strength	0.57 (-0.26, 1.41)	Favours EM	
	Electrophysical modalities vs. manual therapy	3 (124)	Pain (VAS)	0.19 (-2.39, 2.77)	Favours EM	
	Electrophysical modalities vs. manual therapy	3 (234)	Symptom severity (SSS)	1.44 (-0.27, 3.15)	Favours MT	
	Electrophysical modalities vs. manual therapy	3 (234)	Functional status (FSS)	0.99 (0.10, 1.89)	Favours MT	
	Electrophysical modalities vs. manual therapy	2 (54)	Sensory latency	-0.48 (-1.74, 0.78)	No	
	Electrophysical modalities vs. manual therapy	3 (194)	Motor latency	-0.47 (-1.51, 0.56)	No	
	Electrophysical modalities vs. manual therapy	2 (170)	Sensory velocity	0.61 (-0.07, 1.30)	No	
	Electrophysical modalities vs. manual therapy	2 (54)	Grip strength	-0.89 (-2.49, 0.71)	No	
Burger 2017 [25]	LLLT vs. placebo or control	9 (312)	Pain, symptom severity, hand function, and grip strength	No meta-analysis conducted.	NR	“ No strong evidence exists concerning the effects of LLLT on CTS in adults Studies that used 780–860 nm Lasers and energy dosages of 9–11 J/cm2 or 10.8 J reported a more favorable outcome for pain, symptom severity, and functional ability as well as grip strength at the end of treatment and short-term follow up.“ pg. 184
Cheung 2020 [28]	LLLT + SP vs. Sham + SP	3 (226)	Pain (VAS)	2.17, p = 0.03	Favours LLLT + SP	“The use of LLLT in addition to splinting for the management of CTS is not recommended, as LLLT offers limited additional benefits over splining alone in terms of pain reduction, reduction of symptom severity or improved functional status” pg. 24
	LLLT + SP vs. SP	2 (105)	Pain (VAS)	1.30, p = 0.19	No	
	LLLT + SP vs. Sham + SP	2 (145)	SSS	0.44, p = 0.19	No	
	LLLT + SP vs. SP	4 (180)	SSS	0.49, p = 0.62	No	
	LLLT + SP vs. Sham + SP	2 (145)	FSS	0.95, p = 0.34	No	
	LLLT + SP vs. SP	4 (180)	FSS	1.08, p = 0.28	No	
Fallah 2017 [43]	LLLT vs. various comparisons	6 (229)	Pain, sensory impairment	No meta-analysis conducted.	NR	“LLLT accelerated the recovery process of neurapraxia and axonotmesis. Neuronal trauma of organs in patients by laser effect resulted in improved motor neuron electrophysiological parameters and improved muscle function, but it had a placebo effect on sensory function of patients. In three studies out of six studies of CTS patients, a similar effect to sensory function of patients was found.” Pg. 725
Franke 2018 [24]	LLT vs. placebo, US, PNF, corticosteroid, TENS	17 (984)	Pain	No meta-analysis conducted.	NR	“Strong evidence was found for the effectiveness of LLT compared with placebo LLT up to and including 5-week follow-up. From 5 weeks onward, these results were not maintained and moderate evidence (at 7-wk follow-up), no evidence (at 3-mo follow-up, and in the long-term), and limited evidence (based on a single study at 6-mo follow-up) were found for the effectiveness for LLT versus placebo. Ultrasound was more effective than LLT in the short (moderate evidence), mid- (limited evidence), and long term (limited evidence). For all other interventions and intervention regimens that were studied and compared in the included RCTs, only limited, conflicting, and no evidence for the effectiveness of LLT was found.“ pg. 1657
Fu 2019 [26]	SWD and MWD vs. US	No meta-analysis conducted	Pain, hand function, electrophysical test	No meta-analysis conducted	Yes	“Shortwave or microwave diathermy produced pain relief and function improvement in patients with CTS.“ pg. 357
Fulop 2010 [35]	LLLT vs. placebo	1(19)	Pain (VAS, McGill, NDI, WOMAC)	0.84 (0.44 to 1.23)	Favours LLLT	“The large effect size (+ 0.84) obtained in this analysis signifies that phototherapy is a highly effective form of treatment for pain relief” pg. 732
Huisstede 2018 [23]	US, ESWT, heat wrap therapy, local microwave hyperthermia, iontophoresis, pulsed radiofrequency, SWD, IFC	No meta-analysis conducted.	CTS symptoms, pain, grip strength, SSS, FSS, patient global assessment, physical global assessment, pinch strength	No meta-analysis conducted.	NR	“… moderate evidence was found for several electrophysical modalities in the short-term (ultrasound vs placebo, ultrasound vs a corticosteroid injection plus a wrist splint, ESWT plus a neutral night wrist splint vs placebo ESWT with the same splint, interferential current vs TENS or a night-only splint, iontophoresis vs phonophoresis at 4wk, continuous shortwave diathermy vs pulsed shortwave diathermy or placebo pulsed shortwave diathermy) and in the midterm (ultrasound vs placebo, ESWT plus a neutral night wrist splint vs placebo ESWT with the same splint). Moreover, no studies reported on the long-term effectiveness of the interventions. However, even though moderate evidence was found for all of the aforementioned interventions, further research is needed to determine optimal treatment parameters for CTS” pg. 1632
Kim 2019 [36]	ESWT vs. steroid injection, wrist splint, sham, dietary supplement (12–24 weeks)	6 (281)	Overall effect	1.45 (0.44 to 2.46, p = 0.005)	Favours ESWT	“Our meta-analysis revealed that ESWT can improve symptoms, functional outcomes, and electrophysiologic parameters in patients with CTS. Further research is needed to confirm the long-term effects and the optimal ESWT protocol for CTS.” pg. 1
	ESWT vs. steroid injection, wrist splint, sham, dietary supplement (12–24 weeks)	6 (281)	Symptoms (pain, numbness, tingling sensation, or weakness)	1.60 (0.49 to 2.74, p = 0.006)	Favours ESWT	
	ESWT vs. steroid injection, wrist splint, sham, dietary supplement (12–24 weeks)	5 (245)	Functional scores (FSS or DASH)	1.56 (0.18 to 2.94, p = 0.027)	Favours ESWT	
	ESWT vs. steroid injection, wrist splint, dietary supplement (12–24 weeks)	4 (161)	EDx motor component	0.94 (0.35 to 1.54, p = 0.002)	Favours ESWT	
	ESWT vs. steroid injection, wrist splint, sham, dietary supplement (12–24 weeks)	5 (221)	EDx sensory component	0.89 (0.16 to 1.62, p = 0.016)	Favours ESWT	
	ESWT vs. sham (14–24 weeks)	2 (120)	Overall effect	3.15 (1.93 to 4.36, p < 0.001)	Favours ESWT	
	ESWT vs. steroid injection (12 weeks)	2 (61)	Overall effect	0.42 (-0.13 to 0.97, p = 0.135)	Favours ESWT	
	ESWT vs. steroid injection, wrist splint, sham (12–14 weeks)	3 (125)	Overall effect	1.75 (-0.15 to 3.66, p = 0.072)	Favours ESWT	
	ESWT vs. dietary supplement and steroid injection (12–24 weeks)	3 (208)	Overall effect	1.16 (-0.12 to 2.44, p = 0.076)	Favours ESWT	
Li 2020 [37]	ESWT vs. local steroid injection	5 (202)	Pain (VAS)	-0.22 (-1.16 to 0.72, p = 0.65)	No	“In terms of pain relief and function improvement, the effects of ESWT and LCI are not significantly different. In terms of electrophysiological parameters, LCI has a stronger effect on shortening motor distal latency. ESWT is superior to LCI in improving action potential amplitude. ESWT is a non-invasive treatment with fewer complications and greater patient safety. In light of the heterogeneity and limitations, these conclusions require further research for definitive conclusions to be drawn.” Pg. 1
	ESWT vs. local steroid injection	3 (131)	BCTQ	-5.69 (-1.71 to 1.11, p = 0.14)	No	
	ESWT vs. local steroid injection	3 (108)	Sensory distal latency	0.18 (− 0.62 to 0.97, p = 0.66)	No	
	ESWT vs. local steroid injection	5 (201)	Motor distal latency	0.17 (0.10 to 0.25, p < 0.00001)	Favours local steroid injection	
	ESWT vs. local steroid injection	5 (201)	CMAP amplitude	-0.48 (-0.61 to -0.35 p < 0.00001)	Favours ESWT	
	ESWT vs. local steroid injection	3 (108)	SNAP amplitude	-1.56 (-2.62 to -0.50, p = 0.004)	Favours ESWT	
	ESWT vs. local steroid injection	2 (71)	NCV of sensory nerve	-2.33 (-4.77 to 0.11, p = 0.06)	No	
Li 2016 [38]	LLLT vs. placebo	3 (NR)	Motor distal latency short	-0.07 (-0.34 to 0.20), p = 0.91	No	“This study revealed that low-level laser improves hand grip, VAS, and SNAP after 3 months of follow-up for mild to moderate CTS. More high-quality studies using the same laser intervention protocol are needed to confirm the effects of low-level laser in the treatment of CTS.” pg. 1
	LLLT vs. placebo	3 (NR)	Motor distal latency long	-0.61 (-1.89 to 0.65), p = 0.34	No	
	LLLT vs. placebo	2 (NR)	Sensory distal latency short	-0.03 (-0.25 to 0.18), p = 0.75	No	
	LLLT vs. placebo	2 (NR)	Sensory distal latency long	-0.06 (-0.33 to 0.21), p = 0.67	No	
	LLLT vs. placebo	3 (NR)	CMAP long	-0.51 (-1.58, 0.57), p = 0.35	No	
	LLLT vs. placebo	3 (NR)	SNAP long	1.08 (0.44, 1.73), p = 0.001	Favours LLLT	
	LLLT vs. placebo	2 (NR)	Motor nerve velocity short	-0.58 (-2.73, 1.56), p = 0.59	No	
	LLLT vs. placebo	2 (NR)	Sensory nerve velocity long	1.31, (-0.56, 3.18), p = 0.17	No	
	LLLT vs. placebo	5 (NR)	Hand grip (short)	1.46 (-0.85, 3.77), p = 0.22	No	
	LLLT vs. placebo	3 (NR)	Hand grip (long)	0.98 (0.59, 1.37), p < 0.001	Favours LLLT	
	LLLT vs. placebo	4 (NR)	VAS (short)	-0.02 (-2.63, 2.58), p = 0.99	No	
	LLLT vs. placebo	2 (NR)	VAS (long)	0.97 (0.84, 1.11), p < 0.001	Favours LLLT	
	LLLT vs. placebo	4 (NR)	SSS (short)	-1.40 (-8.15, 5.34), p = 0.68	No	
	LLLT vs. placebo	3 (NR)	SSS (long)	0.11 (-0.36, 0.58), p = 0.65	No	
	LLLT vs. placebo	4 (NR)	FSS (short)	-1.58 (-3.29, 0.13), p = 0.07	No	
	LLLT vs. placebo	3 (NR)	FSS (long)	-0.05 (-0.44, 0.35), p = 0.81	No	
Page 2013 [29]	US vs. placebo, another type of US, non-surgical intervention, multicomponent intervention (exercise and splint)	11 (414)	Global rating of improvement, satisfaction with treatment, motor distal latency	No meta-analysis conducted.	N/A	“There is only poor-quality evidence from very limited data to suggest that therapeutic ultrasound may be more effective than placebo for either short- or long-term symptom improvement in people with carpal tunnel syndrome” pg. 21
Rankin 2017 [39]	LLLT vs. placebo	7 (327)	SSS, short-term	-0.36 (-0.78, 0.06), p = 0.09	No	“The evidence is of very low quality, and we found no data to support any clinical effect of LLLT in treating CTS. Only VAS pain and finger pinch strength met previously published MCIDs, but these are likely to be overestimates of effect given the small studies and significant risk of bias. There is low or very low-quality evidence to suggest that LLLT is less effective than ultrasound in the management of CTS based on short-term, clinically significant improvements in pain and finger-pinch strength. There is insufficient evidence to support LLLT being better or worse than any other type of non-surgical treatment in the management of CTS. Any further research of LLLT should be definitive, blinded, and of high quality.” Pg.2
	LLLT vs. placebo	5 (159)	FSS	-0.56 (-1.03, -0.09), p = 0.02	Favours LLLT	
	LLLT vs. placebo	7 (392)	VAS pain	-1.47 (-2.36, -0.58), p = 0.00	Favours LLLT	
	LLLT vs. placebo	5 (286)	Grip strength	2.58 (1.22, 3.95), p = 0.00	Favours LLLT	
	LLLT vs. placebo	2 (121)	Finger-pinch strength	0.94 (0.43, 1.44), p = 0.00	Favours LLLT	
	LLLT vs. placebo	7 (446)	Motor nerve latency	-0.09 (-0.16, -0.03), p = 0.00	Favours LLLT	
	LLLT vs. placebo	5 (307)	Sensory nerve latency	-0.10 (-0.15, -0.06), p < 0.0001	Favours LLLT	
	LLLT vs. placebo	2 (139)	Sensory nerve velocity	1.48 (-5.68, 8.65), p = 0.68	No	
	LLLT vs. US	2 (127)	SSS	0.43 (0.36, 0.50), p < 0.0001	Favours LLLT	
	LLLT vs. US	2 (127)	FSS	0.35 (0.29, 0.41), p < 0.0001	Favours LLLT	
	LLLT vs. US	33 (177)	VAS pain	2.81 (1.21, 4.40), p = 0.00	Favours LLLT	
	LLLT vs. US	2 (77)	Grip strength	-0.89 (-4.30, 2.52), p = 0.61	No	
	LLLT vs. US	33 (177)	Motor nerve latency	0.61 (0.27, 0.95), p = 0.00	Favours LLLT	
	LLLT vs. US	3 (177)	Sensory nerve latency	0.43 (-0.01, 0.87), p = 0.05	Favours LLLT	
	LLLT vs. US	2 (77)	Motor amplitude	-1.90 (-3.63, -0.18), p = 0.03	Favours LLLT	
	LLLT vs. US	3 (NR)	Sensory latency – pre-analysis	0.43 (-0.01, 0.87), p = 0.05	Favours LLLT	
	LLLT vs. US	3 (NR)	Sensory latency, intra-cluster correlation coefficient	0.47 (0.39, 0.55), p < 0.0001	Favours LLLT	
	LLLT vs. steroid injection	2 (73)	Motor latency	-0.04 (-0.39, 0.30), p = 0.8	No	
Robertson 2001 [41]	US vs. placebo US	1 (NR)	Subjective symptom score for main complaint and sensory loss; electroneurographic measurements of median nerve; physical function levels, including strength of handgrip and of finger pinch	No meta-analysis conducted.	NR	“Of these RCTs, the results of 2 trials suggest that therapeutic ultrasound is more effective in treating some clinical problems (carpal tunnel syndrome and calcific tendinitis of the shoulder) than placebo ultrasound, and …” pg. 1339
Roll 2017 [42]	LLLT, US, heat wrap, phonophoresis, iontophoresis	5 (NR)	Clinical outcome	No meta-analysis conducted.	No meta-analysis conducted.	“The evidence for use of any physical agent modality for treatment of CTS is limited; this evidence partially supports the use of US for short-term improvements, but the effectiveness of LLLT and other modalities is not supported.” pg. 8
Xie 2022 [40]	ESWT vs. any non-surgical intervention	7 (291)	Pain	-0.60 (-1.16, -0.05), p = 0.03	Favours ESWT	“The shock wave therapy was observed to have a significant effect on pain relief (MD: 0.60, 95% CI: 1.16 to 0.05, p.0.03), syndrome alleviation (MD: 2.26, 95% CI: 3.24 to 1.27, p < 0.00001) and functional recovery (MD: 1.25 95% CI: 2.08 to 0.43, p.0.003) among the carpal tunnel syndrome patients. As revealed by the subgroup analysis, radial shock wave therapy made a significant difference in pain relief, syndrome alleviation, and functional recovery (p < 0.05). Focused shock wave had no significant effect on pain relief, syndrome alleviation, and functional recovery (p > 0.05).” pg. 177
	ESWT vs. any non-surgical intervention	8 (428)	SSS	-2.26 (-3.24, -1.27), p < 0.00001	Favours ESWT	
	ESWT vs. any non-surgical intervention	8 (428)	FSS	-1.25 (-2.08, -0.43), p = 0.003	Favours ESWT	

*List of abbreviations*: BCTQ, Boston Carpal Tunnel Questionnaire; CMAP, Compound Muscle Action Potential; EDx, electrodiagnosis; EM, electrophysical modalities; ESWT, extracorporeal shockwave therapy; FSS, Functional Status Scale; IFC, interferential currents; Md, mean difference; LCI, local corticosteroid injection; LLLT, low-level laser therapy; MCID, minimal clinically important differences; MT, manual therapy; df, degrees of freedom; MWD, microwave diathermy; N/A, not applicable; NR, not reported; SNAP Sensory Nerve Action Potential; SP, splinting; SSS, Symptom Severity Scale; SWD, short-wave diathermy; US, ultrasound; VAS, visual analogue scale.

#### Light agents: LLLT, non-laser light therapy

Low-level laser therapy was the most frequently assessed intervention, as assessed by 10 of the included SRs [24, 25, 27, 28, 34, 35, 38, 39, 42, 43]. Among these papers, eight SRs addressed only LLLT [24, 25, 27, 28, 35, 38, 39, 43], and two addressed other types of biophysical interventions as well [34, 42]. Out of these 10 SRs, six conducted a meta-analysis [27, 28, 34, 35, 38, 39]. These 10 SRs all had critically low quality according to AMSTAR-2.

Favourable evidence: Six SRs with critically low quality reported beneficial effect of LLLT compared to placebo or manual therapy in pinch or grip strength, symptom severity or functional status of CTS population [24, 25, 27, 34, 35, 49]. Burger et al. specified the effectiveness of LLLT to “studies that used 780–860 nm Lasers and energy dosages of 9–11 J/cm2 or 10.8 J” for pain reduction, symptom severity, functional status, and grip strength [25]. Only one study supported long-term (3 months follow-up) effectiveness of LLLT on hand grip, VAS, and Sensory Nerve Action Potential in mild to moderate CTS, which was mainly according to only one primary study [38]. Fallah et al. which assessed LLLT effectiveness in ‘peripheral somatosensory neuropathy population’, reported that “LLLT accelerated the recovery process of neurapraxia and axonotmesis, improved motor neuron electrophysiological parameters and improved muscle function, it had a placebo effect on sensory function of patients” [43].

Unfavourable evidence: Four SRs with critically low quality reported no benefit of LLLT compared to placebo, splint, US, or other interventions, in pain reduction, functional status improvement, and other electrophysiological measures (sensory and motor distal latencies, and Compound Muscle Action Potential) [24, 27, 39, 42]. Two of these studies specifically reported that there was no evidence on the long-term effectiveness of LLLT [24, 27]. Cheung et al. reported that comparing LLLT + splint to splint alone, LLLT does not provide any additional benefit [28]. For non-laser light therapy, two SRs reported on the same primary study on polarized polychromatic noncoherent light therapy (PPNL) [23, 34]. According to their results, no evidence was found for the effectiveness of PPNL in short-term improvement of pain or disease severity [23, 34].

#### ESWT

Extracorporeal shockwave therapy was assessed in five SRs [23, 34, 36, 37, 40], of which four did a meta-analysis [34, 36, 37, 40]. Four of these SRs had a critically low quality [23, 34, 36, 40], and one had a low quality [37]. The population was people with radial, ulnar, and median neuropathies in one SR [34], and only CTS in the remaining four SRs [23, 36, 37, 40].

Favourable evidence: Four studies with critically low quality consistently concluded that ESWT (plus splint) could improve symptoms, functional parameters, and some electrophysiologic parameters in patients with mild or moderate CTS in short and mid-term [23, 34, 36, 40]. Li et al. reported the improvement of Compound Muscle Action Potential, mean difference = -0.48 (95% CI -0.61 to -0.35 p < 0.00001) and Sensory Nerve Action Potential amplitudes, mean difference = -1.56 (95% CI -2.62 to -0.50, p = 0.004) following the use of ESWT versus local steroid injections [37].

Unfavourable evidence: Li et al. reported no difference in pain, Boston Carpal Tunnel Questionnaire, sensory distal latency, or nerve conduction velocity of ESWT compared to local steroid injection [37]. Further, they reported superior results in improving motor distal latency for local steroid injection, but the effect size was small, mean difference = 0.17 (0.10 to 0.25, p < 0.00001) [37]. No studies reported the long-term effectiveness of ESWT.

#### Ultrasound

Ultrasound was assessed by five SRs [23, 29, 34, 41, 42], of which two conducted meta-analysis [29, 34]. Except for one SR with low quality [29], the remaining four SRs had critically low qualities [23, 34, 41, 42]. Among these five SRs, two were specifically on CTS population [23, 29], one was on people with radial, ulnar, and median neuropathies [34], and two were on adults with MSK disorders of the forearm, wrist, and hand [41, 42].

Favourable evidence: all five SRs consistently reported the beneficial effect of ultrasound in improving symptom severity, functional status, pain, global rating of improvement, satisfaction with treatment, and other electrophysiological measures (sensory and motor distal latencies) compared to manual therapy [34], or placebo [23, 29, 41, 42]. Huisstede et al. 2018 reported short-term effectiveness of ultrasound compared to placebo or corticosteroid injection plus a wrist splint, and mid-term effectiveness of ultrasound compared to placebo in CTS population [23]. Even though these five SRs used different tools to assess the ROB or quality of the primary studies, they all reported the quality of the primary studies to be low or very low.

Unfavourable evidence: there was no unfavourable evidence against the use of ultrasound in the CTS population.

#### MWD or SWD

Overall, three SRs, with critically low quality assessed SWD [23, 26, 34], and one SR with critically low quality assessed MWD [26]. Among these SRs, one did a meta-analysis for SWD [34]. The findings of all SRs were from low or unclear quality or at ROB primary studies. One study was on median, ulnar, or radial nerve population [34], one study was on peripheral nerve injuries [26], and one on CTS [50].

Favorable evidence: Huisstede et al. reported short-term effectiveness of continuous SWD versus pulsed SWD, or placebo pulsed SWD [23]. Fu et al. reported improvements in pain, hand function, and electrophysiological parameters with using SWD according to three RCTs [26]. Further, they reported improvement in pain and hand function with no change in electrophysiological parameters with MWD according to one RCT [26].

Unfavourable evidence: Bula-Oyola et al. (critically low-quality SR) with two primary RCTs found no evidence for the effectiveness of SWD for CTS management either in short or long-term [34].

#### Athermal agents: magnetic field therapy and pulsed radiofrequency

Two forms of athermal agents were assessed in people with CTS, magnetic field therapy (n = 2 studies) and pulsed radiofrequency (n = 1 study) [23, 34]. Both SRs were of critically low quality and both the intervention were assessed in a limited number of primary studies.

Favourable evidence: no favourable evidence was found on the effectiveness of magnetic field therapy (statis, dynamic, or pulsed) in the short or long term. For pulsed radiofrequency, Huisstede et al. included one high-quality RCT which assessed pulsed radiofrequency as additive to wrist splint [23]. They reported “there is moderate evidence for 1 session of ultrasound-guided pulsed radiofrequency added to a splinting regimen in the short term.” [23]

Unfavourable evidence: Two SRs found limited and conflicting evidence on the effectiveness of magnetic field therapy for improving symptoms, function, or electrophysiological parameters. No unfavourable evidence was found for pulsed radiofrequency even though the evidence was very limited.

#### Transdermal drug delivery: phonophoresis and iontophoresis

Transdermal drug delivery was assessed in three SR’s (iontophoresis = 2, phonophoresis = 1) [23, 42]. Both SRs had critically low quality and none were able to perform a meta-analysis. The population was people with MSK disorders of upper limb in the study by Roll and Hardison [42], and CTS in the study by Huisstede et al. [23, 42] The evidence was very limited on the effectiveness of transdermal drug delivery for the management of CTS.

Favourable evidence: Both SRs included the same two primary studies, one with high and another one with low quality. According to the SR by Huisstede et al. “there is moderate evidence in favor of phonophoresis versus 0.4% dexamethasone sodium phosphate or 0.1% betamethasone iontophoresis in the short term.” [23] This was in line with the conclusion of the SR by Roll and Hardison [42].

Unfavourable evidence: no unfavourable evidence was found even though the evidence was very limited.

#### Heat wrap therapy

Heat wrap therapy was studied in two SRs with critically low quality, and no meta-analyses were performed [23, 42].

Favourable evidence: According to both SRs, based on the findings of one RCT with low quality, low-level heat wrap therapy (40 C [104 F]) was more effective in managing pain, stiffness, and grip strength in short term (3-days follow-up) compared to oral placebo [23, 42].

Unfavourable evidence: no unfavourable evidence was found even though the evidence was very limited.

#### Electrical stimulations: interferential currents, TENS

Two types of electrical stimulation were studied in a single SR in people with CTS. The quality was critically low, and no meta-analyses was performed due to the limited number of RCTs [23]. Huisstede et al. reported that there is moderate quality evidence on the short-term effectiveness of interferential currents in improving pain and Boston Carpal Tunnel questionnaire scores when compared to TENS or nightly splinting [23].

### Citation mapping/matrix

We calculated the CCA index for LLLT, ultrasound, ESWT, and SWD/MWD since three or more SRs addressed these interventions. APPENDIX III demonstrates the citation matrix for all the included SRs and their interventions, including interventions with less than three SRs addressing them.

**For LLLT**, there were 10 SRs, 28 primary studies, reported 98 times. Therefore, the CCA index was 70/280, and the overlap of the SRs for LLLT was 25%. Among these SRs, Rankin et al., 2017, was the most comprehensive one which covered 22 of 28 reported primary studies [39].

**For ultrasound**, there were five SRs, 17 primary studies, and reported 29 times. Hence, the CCA index was 12/68 and the overlap was 17% in the SRs. Among these five SRs on the effectiveness of ultrasound on CTS management, Page et al. (2013) was the most comprehensive one (11 primary RCTs) and had the highest quality [29].

**For ESWT**, there were five SRs, 12 primary studies, repeated 29 times. Therefore, the CCA index was 17/48, leading to an overlap of 35%. Among the four SRs, Xie et al., were the most comprehensive one, including six primary studies relating to ESWT for the management of CTS [40].

**For SWD/MWD**, there were three SRs, five primary studies, reported eight times. Based on these, the CCA index was 3/10, and the overlap of the SRs for SWD/MWD was 30%. The study by Fu et al. 2019 was the most comprehensive SR, addressing all the existing primary studies reported by all other SRs, except for one primary study which was published a year later in 2020, and was captured by Bula-Oyola et al., 2021.

## Discussion

This overview identified 17 studies which examined the effectiveness of 12 different biophysical agents for the management of symptoms of individuals with CTS. Overall, there is low to critically low-quality evidence demonstrating clinically important usefulness of LLLT, ultrasound, ESWT, and SWD. The overall quality of the evidence was low to critically low, reflecting lack of protocol establishment prior to the conduct of the study, not reporting on the exclusion reasons, not using a satisfactory technique in assessing the ROB of the primary studies, not accounting for the ROB of the primary studies when conducting a meta-analysis or in discussing their findings. In the following paragraphs we will discuss the findings for the most frequently assessed biophysical agent, in order of frequency.

The findings from the SRs were conflicting regarding the effectiveness of LLLT, which makes sense because the overlap between the primary studies was only 25%. There was low overlap because different SRs had different inclusion and exclusion criteria, or searched different databases, resulting in different primary studies, contributing to the conflicting reports of the SRs. Rankin et al.’s study, which covered 22 (of the total 28 primary studies identified by this overview) and used a validated standardized ROB assessment tool (Cochrane 7-item ROB checklist) [44], reported that 21 studies were at unclear or high ROB [39]. They reported “many were not blinded. The quality of the studies across outcomes for each intervention was largely very low, and any point estimates of effect or harm should be interpreted with great caution. Even without this fact, the effect sizes seen were modest or small and may not have any clinical relevance.” p.29 [39]. One certainty confirmed by all SRs is that there is no solid high-quality evidence on the *long-term* effectiveness of LLLT in management of CTS. Despite some SRs confirming the short-term effectiveness of LLLT, it is unclear whether it is superior to splinting alone, placebo, manual therapy, or other interventions in the long-term.

Therapists have been using ultrasound in managing CTS for a long time, as Watson notes “the use of therapeutic ultrasound as an element of physiotherapy practice is well established, but the nature of that practice has changed significantly over the last 20 years.” p.321 [51]. Overall, based on the included SRs, it appears that ultrasound is potentially an effective biophysical agent in ameliorating CTS symptoms in the short-term, but no dose-response relationship has been identified [29]. Results from Page et al. 2013 and Huisstede et al. 2018 (two studies who only focused on ultrasound and had higher quality) consistently show no difference in one ultrasound regimen being superior to another in managing CTS [23, 29]. Further, the included SRs consistently reported lack of evidence on the mid- and long-term effectiveness of ultrasound. Hence, more high-quality studies are needed to assess long-term effectiveness and a potential dose-response relationship.

The included SRs report potential effectiveness of ESWT in improving CTs symptoms, some electrophysiological parameters, and functional outcomes in the short-term. The findings of the four SRs included in this overview, were based on 11 primary studies, mostly with high or unclear ROB, and the meta-analyses report small effect sizes. When compared with local steroid injections, no superior results were found for ESWT [36, 37]. Similarly, it is unclear if ESWT plus splinting is superior to splinting alone in the long-term [36]. Kim et al. 2019 did a sub-group analysis of the two types of ESWT (radial and focused ESWT) and found no significant difference between them [36].

According to Fu et al. 2019, only a limited number of RCTs focused on the effectiveness of diathermy in the management of peripheral nerve injuries, in particular CTS [26]. Fu et al. reported on four RCTs on this topic, and we found another more recent RCT as captured by Bula-Oyola’s SR [34]. Diathermy is believed to increase the heat in the deep tissue, and leads to increase in soft tissue elasticity, vasodilatation, local blood flow, and decreases the muscle spasm [26]. Given this, despite the fact that diathermy could be a potentially beneficial biophysical agent in CTS, the evidence is scarce; the five primary RCTs each had fewer than 50 participants in each group, with short-term follow-ups.

Other thermal and athermal agents, transdermal drug delivery methods, and electrical stimulation had less evidence and were studied in fewer SRs or primary studies. Even when these modalities were reviewed in two or three SRs, our citation mapping indicated that the findings were based on the same primary studies, therefore, we could not make comparisons among different SRs. In most cases, these primary studies were of low quality and with short follow-up periods.

The results of this overview align with those reported by the American Physical Therapy Association clinical practice guidelines [16]. This guideline advise against using low-level laser therapy or other types of non-laser light therapy, thermal ultrasound, iontophoresis or magnets in the non-surgical management of individuals with CTS. Further, they recommend trialing superficial heat or interferential currents for short-term symptom relief and application of MWD/SWD within non-surgical interventions for individuals with mild to moderate CTS.

### Study limitations

We only included SRs that addressed biophysical agents, and no other types of CTS management techniques, such as exercise, education, or manual therapy. Acknowledging the importance of the other management techniques, we limited the scope of this overview to focus mainly on biophysical agents because of the vast diversity of the available techniques. We believe the clinicians would have a clearer understanding of the biophysical agents when focusing only on this type of intervention. Another limitation was that we may have missed studies due to the extensiveness of the topic and because the search was limited to articles published in peer-reviewed journals. To minimize this risk of publication or language bias, we developed our search strategy in consultation with a health science librarian. Furthermore, we only included published systematic reviews. Studies with positive or significant results are more likely to be published, while studies with negative or non-significant results may be underrepresented. This bias could potentially inflate the reported effectiveness of biophysical agents in the treatment of CTS and introduce a publication bias.

Lastly, one limitation which is inherent to the design of overviews of SRs was that we only relied on the SRs for their conclusion of the primary studies. We did not assess the quality of the primary studies or their findings. This introduces the possibility of misreading by the authors of SRs. Also, some primary studies were included in more than one SR. To address this, we did a citation mapping and added all the primary studies so that readers can easily find evidence on each biophysical agent.

## Conclusion

Biophysical agents are essential tools in managing and improving symptoms related to CTS. The large body of studies found by this overview reflects on the growing importance of these techniques. SWD/ MWD, non-thermal ultrasound, superficial heat, and phonophoresis can be used for the short-term relief of CTS symptoms. However, none of the studied tools were consistently effective for improving CTS symptoms in the long-term. More high-quality RCTs are needed to confirm these findings.

### Electronic supplementary material

Below is the link to the electronic supplementary material.

Supplementary Material 1

## Data Availability

All data generated or analysed during this study are included in this published article [and its supplementary information files].
