# Supplementary material for: The effectiveness of biophysical agents in the treatment of carpal tunnel syndrome- an umbrella review
Source: BMC Musculoskelet Disord. 2023 Aug 10;24:645. doi: 10.1186/s12891-023-06778-z (PMC10416372; doi:10.1186/s12891-023-06778-z)
Supplement: Supplementary file 1 — Supplementary Material 1 [file 12891_2023_6778_MOESM1_ESM.docx]

**Appendices**

**Appendix I- MEDLINE search strategy**

Ovid MEDLINE(R) ALL <1946 to November 19, 2021>

1 consensus development conference/ or meta-analysis/ or "systematic review"/ 259646

2 "review literature as topic"/ or consensus development conferences as topic/ or systematic reviews as topic/ 17968

3 meta-analysis as topic/ or network meta-analysis/ 23461

4 "review"/ 2895180

5 1 or 2 or 3 or 4 3049677

6 median neuropathy/ or carpal tunnel syndrome/ or nerve compression syndromes/ 19323

7 CTS.mp. 10561

8 Carpal Tunnel Syndrome/ or Nerve Compression Syndromes/ or Median Nerve/ 26054

9 Median Neuropathy/pp, pc, rh, th [Physiopathology, Prevention & Control, Rehabilitation, Therapy] 140

10 6 or 7 or 8 or 9 34293

11 Humans/rh [Rehabilitation] 51

12 Carpal Tunnel Syndrome/pp, pc, rh, th [Physiopathology, Prevention & Control, Rehabilitation, Therapy] 3121

13 Self-Help Devices/ 5345

14 Assistive technology.mp. 2369

15 Ergonomics/ or Occupational Health/ 46059

16 orthopedic fixation devices/ or orthotic devices/ or braces/ 17164

17 wrist brace.mp. 35

18 biophysical agents.mp. 6

19 combined modality therapy/ or cryotherapy/ or electric stimulation therapy/ or diathermy/ or photothermal therapy/ or laser therapy/ or magnetic field therapy/ or pain management/ or patient care/ or phototherapy/ or physical therapy modalities/ or placebos/ or radiofrequency therapy/ or rehabilitation/ or therapy, computer-assisted/ 398695

20 dry heat therapy.mp. 1

21 laser therapy/ or physical therapy modalities/ or electric stimulation therapy/ or exercise movement techniques/ or exercise therapy/ or extracorporeal shockwave therapy/ or hydrotherapy/ or musculoskeletal manipulations/ or rehabilitation/ 162146

22 Pain Management/ or Electric Stimulation Therapy/ or Transcutaneous Electric Nerve Stimulation/ or interferential current.mp. 62123

23 ES.mp. 71931

24 electromagnetic fields/ or electromagnetic radiation/ or microwaves/ 36205

25 diathermy/ or short-wave therapy/ or ultrasonic therapy/ 13454

26 shortwave diathermy.mp. 91

27 Laser Therapy/ or non-laser light therapy.mp. or Low-Level Light Therapy/ 45449

28 Anti-Inflammatory Agents, Non-Steroidal/ or Administration, Topical/ or topical anti-inflammatory drugs.mp. or Anti-Inflammatory Agents/ 187323

29 Phonophoresis/ or Iontophoresis/ 7735

30 manual therapy.mp. or Musculoskeletal Manipulations/ 3999

31 Therapy, Soft Tissue/ or soft tissue mobilization.mp. or Massage/ 6776

32 joint mobilization.mp. 473

33 tendon gliding.mp. 243

34 Median Nerve/ or neurodynamic.mp. 10366

35 muscle stretching exercises/ or occupational therapy/ or telerehabilitation/ or physical therapy modalities/ or exercise movement techniques/ or exercise therapy/ 95810

36 11 or 12 or 13 or 14 or 15 or 16 or 17 or 18 or 19 or 20 or 21 or 22 or 23 or 24 or 25 or 26 or 27 or 28 or 29 or 30 or 31 or 32 or 33 or 34 or 35 845850

37 5 and 10 and 36 923

**Appendix II- Exclusion reasons after full-text review**

| **Title** | **Authors** | **Published Year** | **Journal** | **DOI** | **Exclusion reasons** |
| --- | --- | --- | --- | --- | --- |
| Application of ESWT in post-operative treatment in Carpal Tunnel Syndrome - a review. | Ambroziak, Maciej | 2020 | Polski przeglad chirurgiczny | https://dx.doi.org/10.5604/01.3001.0014.0947 | Not SYSTEMATIC review |
| Carpal Tunnel Syndrome and Meralgia Paresthetica in Pregnancy. | Gooding, Megan S; Evangelista, Victor; Pereira, Leonardo | 2020 | Obstetrical & gynecological survey | https://dx.doi.org/10.1097/OGX.0000000000000745 | Not SYSTEMATIC review |
| Recent Advances in the Understanding and Management of Carpal Tunnel Syndrome: a Comprehensive Review. | Urits, Ivan; Gress, Kyle; Charipova, Karina; Orhurhu, Vwaire; Kaye, Alan D; Viswanath, Omar | 2019 | Current pain and headache reports | https://dx.doi.org/10.1007/s11916-019-0811-z | Not SYSTEMATIC review |
| Acupuncture and Related Interventions for the Treatment of Symptoms Associated with Carpal Tunnel Syndrome: Summary of a Cochrane Review. | Wieland, L Susan | 2019 | Explore (New York, N.Y.) | https://dx.doi.org/10.1016/j.explore.2019.02.008 | Not SYSTEMATIC review |
| Conservative treatment in patients with mild to moderate carpal tunnel syndrome: A systematic review. | Jimenez Del Barrio, S; Bueno Gracia, E; Hidalgo Garcia, C; Estebanez de Miguel, E; Tricas Moreno, J M; Rodriguez Marco, S; Ceballos Laita, L | 2018 | Neurologia (Barcelona, Spain) | https://dx.doi.org/10.1016/j.nrl.2016.05.018 | Not English |
| Guiding Treatment for Carpal Tunnel Syndrome. | Wang, Leilei | 2018 | Physical medicine and rehabilitation clinics of North America | https://dx.doi.org/10.1016/j.pmr.2018.06.009 | Not SYSTEMATIC review |
| Rehabilitation following carpal tunnel release: A Cochrane review summary. | Whitehead, Lisa | 2018 | International journal of nursing studies | https://dx.doi.org/10.1016/j.ijnurstu.2017.08.012 | Wrong study design |
| Carpal Tunnel Syndrome: Making Evidence-Based Treatment Decisions. | Calandruccio, James H; Thompson, Norfleet B | 2018 | The Orthopedic clinics of North America | https://dx.doi.org/10.1016/j.ocl.2017.11.009 | Not SYSTEMATIC review |
| Treatment of carpal tunnel syndrome : from ultrasonography to ultrasound guided carpal tunnel release. | Petrover, David; Richette, Pascal | 2018 | Joint bone spine | https://dx.doi.org/10.1016/j.jbspin.2017.11.003 | Wrong intervention |
| Conservative therapeutic management of carpal tunnel syndrome. | Martins, Roberto Sergio; Siqueira, Mario Gilberto | 2017 | Arquivos de neuro-psiquiatria | https://dx.doi.org/10.1590/0004-282X20170152 | Not SYSTEMATIC review |
| Carpal Tunnel Syndrome: Symptoms, Causes and Treatment Options. Literature Reviev. | Zamborsky, Radoslav; Kokavec, Milan; Simko, Lukas; Bohac, Martin | 2017 | Ortopedia, traumatologia, rehabilitacja | https://dx.doi.org/10.5604/15093492.1232629 | Not SYSTEMATIC review |
| Efficacy, safety, and cost of surgical versus nonsurgical treatment for carpal tunnel syndrome: A systematic review and meta-analysis. | Ren, Yi-Ming; Wang, Xi-Shan; Wei, Zhi-Jian; Fan, Bao-You; Lin, Wei; Zhou, Xian-Hu; Feng, Shi-Qing | 2016 | Medicine | https://dx.doi.org/10.1097/MD.0000000000004857 | Retracted |
| Carpal Tunnel Syndrome: Diagnosis and Management. | Wipperman, Jennifer; Goerl, Kyle | 2016 | American family physician |  | Not SYSTEMATIC review |
| Carpal tunnel syndrome: clinical features, diagnosis, and management. | Padua, Luca; Coraci, Daniele; Erra, Carmen; Pazzaglia, Costanza; Paolasso, Ilaria; Loreti, Claudia; Caliandro, Pietro; Hobson-Webb, Lisa D | 2016 | The Lancet. Neurology | https://dx.doi.org/10.1016/S1474-4422(16)30231-9 | Not SYSTEMATIC review |
| Acute Carpal Tunnel Syndrome: A Review of Current Literature. | Gillig, Jonathan D; White, Stephen D; Rachel, James Nicholas | 2016 | The Orthopedic clinics of North America | https://dx.doi.org/10.1016/j.ocl.2016.03.005 | Not SYSTEMATIC review |
| Carpal and cubital tunnel and other, rarer nerve compression syndromes. | Assmus, Hans; Antoniadis, Gregor; Bischoff, Christian | 2015 | Deutsches Arzteblatt international | https://dx.doi.org/10.3238/arztebl.2015.0014 | Not SYSTEMATIC review |
| Diagnosing and managing carpal tunnel syndrome in primary care. | Burton, Claire; Chesterton, Linda S; Davenport, Graham | 2014 | The British journal of general practice : the journal of the Royal College of General Practitioners | https://dx.doi.org/10.3399/bjgp14X679903 | Not SYSTEMATIC review |
| Rehabilitation following carpal tunnel release. | Peters, Susan; Page, Matthew J; Coppieters, Michel W; Ross, Mark; Johnston, Venerina | 2013 | The Cochrane database of systematic reviews | https://dx.doi.org/10.1002/14651858.CD004158.pub2 | Outdated SR |
| Cochrane corner: ergonomic positioning or equipment for treating carpal tunnel syndrome. | Buchan, S; Amirfeyz, R | 2013 | The Journal of hand surgery, European volume | https://dx.doi.org/10.1177/1753193413478507 | Not SYSTEMATIC review |
| Effects of sensory reeducation programs on functional hand sensibility after median and ulnar repair: a systematic review. | Miller, Leanne K; Chester, Rachel; Jerosch-Herold, Christina | 2012 | Journal of hand therapy : official journal of the American Society of Hand Therapists | https://dx.doi.org/10.1016/j.jht.2012.04.001 | Wrong patient population |
| Evaluation and treatment of failed carpal tunnel release. | Neuhaus, Valentin; Christoforou, Dimitrios; Cheriyan, Thomas; Mudgal, Chaitanya S | 2012 | The Orthopedic clinics of North America | https://dx.doi.org/10.1016/j.ocl.2012.07.013 | Wrong intervention |
| Osteopathic manipulative medicine for carpal tunnel syndrome. | Siu, Gilbert; Jaffe, J Douglas; Rafique, Maryum; Weinik, Michael M | 2012 | The Journal of the American Osteopathic Association |  | Not SYSTEMATIC review |
| Is surgical intervention more effective than non-surgical treatment for carpal tunnel syndrome? A systematic review. | Shi, Qiyun; MacDermid, Joy C | 2011 | Journal of orthopaedic surgery and research | https://dx.doi.org/10.1186/1749-799X-6-17 | Wrong intervention |
| Diagnosis, treatment and follow-up of the carpal tunnel syndrome: a review. | Alfonso, Calogero; Jann, Stefano; Massa, Roberto; Torreggiani, Aldo | 2010 | Neurological sciences : official journal of the Italian Neurological Society and of the Italian Society of Clinical Neurophysiology | https://dx.doi.org/10.1007/s10072-009-0213-9 | Not SYSTEMATIC review |
| Carpal tunnel syndrome. | Ashworth, Nigel L | 2011 | BMJ clinical evidence |  | Not SYSTEMATIC review |
| Carpal tunnel syndrome. | Patijn, Jacob; Vallejo, Ricardo; Janssen, Markus; Huygen, Frank; Lataster, Arno; van Kleef, Maarten; Mekhail, Nagy | 2011 | Pain practice : the official journal of World Institute of Pain | https://dx.doi.org/10.1111/j.1533-2500.2011.00457.x | Not SYSTEMATIC review |
| Workplace management of upper limb disorders: a systematic review. | Dick, F D; Graveling, R A; Munro, W; Walker-Bone, K; Guideline Development Group | 2011 | Occupational medicine (Oxford, England) | https://dx.doi.org/10.1093/occmed/kqq174 | Wrong intervention |
| Current concepts of carpal tunnel syndrome: pathophysiology, treatment, and evaluation. | Uchiyama, Shigeharu; Itsubo, Toshirou; Nakamura, Koichi; Kato, Hiroyuki; Yasutomi, Takashi; Momose, Toshimitsu | 2010 | Journal of orthopaedic science : official journal of the Japanese Orthopaedic Association | https://dx.doi.org/10.1007/s00776-009-1416-x | Not SYSTEMATIC review |
| Evidence supporting the use of physical modalities in the treatment of upper extremity musculoskeletal conditions. | Valen, Peter A; Foxworth, Judy | 2010 | Current opinion in rheumatology | https://dx.doi.org/10.1097/BOR.0b013e328335a851 | Not SYSTEMATIC review |
| Interventions for recurrent/persistent carpal tunnel syndrome after carpal tunnel release. | Amadio, Peter C | 2009 | The Journal of hand surgery | https://dx.doi.org/10.1016/j.jhsa.2009.04.031 | Not SYSTEMATIC review |
| Management of carpal tunnel syndrome. | Anonymous | 2009 | Drug and therapeutics bulletin | https://dx.doi.org/10.1136/dtb.2009.07.0028 | No full-text |
| Surgical versus non-surgical treatment for carpal tunnel syndrome. | Verdugo, Renato J; Salinas, Rodrigo A; Castillo, Jose L; Cea, Jose G | 2008 | The Cochrane database of systematic reviews | https://dx.doi.org/10.1002/14651858.CD001552.pub2 | Wrong intervention |
| Carpal tunnel syndrome. | Ashworth, Nigel L | 2007 | BMJ clinical evidence |  | Duplicate |
| Carpal tunnel syndrome--a comprehensive review. | Haase, J | 2007 | Advances and technical standards in neurosurgery |  | Book chapter |
| Carpal tunnel syndrome. | Cranford, C Sabin; Ho, Jason Y; Kalainov, David M; Hartigan, Brian J | 2007 | The Journal of the American Academy of Orthopaedic Surgeons |  | Not SYSTEMATIC review |
| Carpal tunnel syndrome. | Bland, Jeremy D P | 2007 | BMJ (Clinical research ed.) |  | Not SYSTEMATIC review |
| Treatment of carpal tunnel syndrome. | Bland, Jeremy D P | 2007 | Muscle & nerve |  | Not SYSTEMATIC review |
| Ergonomic and physiotherapeutic interventions for treating work-related complaints of the arm, neck or shoulder in adults. | Verhagen, A P; Karels, C; Bierma-Zeinstra, S M A; Burdorf, L; Feleus, A; Dahaghin, S; de Vet, H C W; Koes, B W | 2006 | The Cochrane database of systematic reviews |  | Withdrawn |
| Photobiomodulation of pain in carpal tunnel syndrome: review of seven laser therapy studies. | Naeser, Margaret A | 2006 | Photomedicine and laser surgery |  | Wrong study design |
| [Treatment of carpal tunnel syndrome]. | Ly-Pen, Domingo; Andreu, Jose Luis | 2005 | Medicina clinica |  | No full-text |
| Carpal tunnel syndrome. | Bland, Jeremy D P | 2005 | Current opinion in neurology |  | Duplicate |
| Conservative interventions for carpal tunnel syndrome. | Michlovitz, Susan L | 2004 | The Journal of orthopaedic and sports physical therapy |  | Not SYSTEMATIC review |
| Ergonomic and physiotherapeutic interventions for treating upper extremity work related disorders in adults. | Verhagen, A P; Bierma-Zeinstra, S M A; Feleus, A; Karels, C; Dahaghin, S; Burdorf, L; de Vet, H C W; Koes, B W | 2004 | The Cochrane database of systematic reviews |  | Withdrawn |
| Primary care management of carpal tunnel syndrome. | Burke, F D; Ellis, J; McKenna, H; Bradley, M J | 2003 | Postgraduate medical journal |  | Not SYSTEMATIC review |
| Surgical versus non-surgical treatment for carpal tunnel syndrome. | Verdugo, R J; Salinas, R S; Castillo, J; Cea, J G | 2003 | The Cochrane database of systematic reviews |  | Wrong intervention |
| Management of carpal tunnel syndrome. | Viera, Anthony J | 2003 | American family physician |  | Not SYSTEMATIC review |
| A review of treatment for carpal tunnel syndrome. | Wilson, J K; Sevier, T L | 2003 | Disability and rehabilitation |  | Not SYSTEMATIC review |
| Carpal tunnel syndrome: current theory, treatment, and the use of B6. | Holm, Gregory; Moody, Linda E | 2003 | Journal of the American Academy of Nurse Practitioners |  | Narrative review |
| [Recommended management of carpal tunnel syndrome. First wrist splinting, surgery in sensory deficit/atrophy]. | Atroshi, Isam | 2003 | Lakartidningen |  | No full-text |
| Management of carpal tunnel syndrome in the working population. | Kasdan, Morton L; Lewis, Kathleen | 2002 | Hand clinics |  | Not SYSTEMATIC review |
| Enabling meta-analysis in systematic reviews on carpal tunnel syndrome. | Gerritsen, Annette A M; de Vet, Henrica C W; Scholten, Rob J P M; van Tulder, Maurits W; Bouter, Lex M | 2002 | The Journal of hand surgery |  | Wrong intervention |
| Carpal tunnel syndrome. | Sen, D; Chhaya, S; Morris, V H | 2002 | Hospital medicine (London, England : 1998) |  | Not SYSTEMATIC review |
| Repetitive motion hand disorders. | Chin, Douglas H C L; Jones, Neil F | 2002 | Journal of the California Dental Association |  | Wrong patient population |
| Carpal tunnel syndrome: conservative and nonconservative treatment. A chiropractic physician's perspective. | Davis, P T; Hulbert, J R | 1998 | Journal of manipulative and physiological therapeutics |  | Not SYSTEMATIC review |
| Carpal tunnel syndrome: a review. | Cantatore, F P; Dell'Accio, F; Lapadula, G | 1997 | Clinical rheumatology |  | Outdated SR |
| Carpal tunnel syndrome. | von Schroeder, H P; Botte, M J | 1996 | Hand clinics |  | Outdated SR |
| [The carpal tunnel syndrome. The clinical and therapeutic aspects]. | Ruiz Martin, J | 1994 | Atencion primaria |  | Not English |
| Therapeutic techniques to enhance nerve gliding in thoracic outlet syndrome and carpal tunnel syndrome. | Totten, P A; Hunter, J M | 1991 | Hand clinics |  | Book chapter |
| Carpal tunnel syndrome: a review. | Dorwart, B B | 1984 | Seminars in arthritis and rheumatism |  | Not SYSTEMATIC review |
| Conservative management of carpal tunnel syndrome | Burke, D.T. | 1997 | Physical Medicine and Rehabilitation Clinics of North America | http://dx.doi.org/10.1016/s1047-9651%2818%2930314-0 | Not SYSTEMATIC review |
| Upper Limb Interventions | Chianca, Vito; Pozzi, Grazia; Sconfienza, Luca Maria; Messina, Carmelo; Albano, Domenico; Bazzocchi, Alberto | 2019 | Radiologic Clinics of North America | http://dx.doi.org/10.1016/j.rcl.2019.05.002 | Not SYSTEMATIC review |
| Effectiveness of surgical versus conservative treatment for carpal tunnel syndrome: A systematic review, meta-analysis and qualitative analysis | Klokkari, Diony; Mamais, Ioannis | 2018 | Hong Kong Physiotherapy Journal | http://dx.doi.org/10.1142/S1013702518500087 | Wrong intervention |
| Rehabilitation following carpal tunnel release | Peters, Susan; Page, Matthew J; Coppieters, Michel W; Ross, Mark; Johnston, Venerina | 2013 | The Cochrane database of systematic reviews |  | Duplicate |
| Acute carpal tunnel syndrome in trauma | Jhattu, Hardeep; Klaassen, Sophie; Ying, Charlotte; Ali Hussain, Muhammad | 2012 | European Journal of Plastic Surgery | http://dx.doi.org/10.1007/s00238-012-0732-0 | Wrong intervention |
| The effectiveness of particular physiotherapy techniques in the treatment of carpal tunnel syndrome - Application of low-level laser therapy based on a review of the literature | Bartkowiak, Zuzanna; Zgorzalewicz-Stachowiak, Malgorzata; Nowicka, Anna | 2011 | Fizjoterapia | http://dx.doi.org/10.2478/v10109-011-0026-2 | Not SYSTEMATIC review |
| Current concepts in carpal tunnel syndrome: A review of the literature | Lewis, C.; Mauffrey, C.; Newman, S.; Lambert, A.; Hull, P. | 2010 | European Journal of Orthopaedic Surgery and Traumatology | http://dx.doi.org/10.1007/s00590-010-0585-9 | Not SYSTEMATIC review |
| Management of carpal tunnel syndrome | Jones, Melissa C. | 2010 | U.S. Pharmacist |  | Not SYSTEMATIC review |
| Current options for nonsurgical management of carpal tunnel syndrome | Carlson, Hans; Colbert, Agatha; Frydl, Jennifer; Arnall, Elizabeth; Elliott, Molly; Carlson, Nels | 2010 | International Journal of Clinical Rheumatology | http://dx.doi.org/10.2217/ijr.09.63 | Not SYSTEMATIC review |
| Systematic review of phisiotherapy treatments with better evidence for the carpal tunnel syndrome | Alvayay, C.S.; Arce, A. | 2008 | Revista de la Sociedad Espanola del Dolor |  | Not English |
| Braces and splints for musculoskeletal conditions | Van Durme, Daniel J.; Gravlee, Jocelyn R. | 2007 | American Family Physician |  | Not SYSTEMATIC review |
| The management of carpal tunnel syndrome | Hasham, S.; Burke, F.D. | 2005 | Minerva Ortopedica e Traumatologica |  | Not SYSTEMATIC review |
| Treatment options for carpal tunnel syndrome | Hui, Andrew C.F.; Wong, Shiu-Man | 2005 | Therapy | http://dx.doi.org/10.1586/14750708.2.3.455 | Not SYSTEMATIC review |
| Treatment of carpal tunnel syndrome: A review of the non-surgical approaches with emphasis in neural mobilization | Kostopoulos, Dimitrios | 2004 | Journal of Bodywork and Movement Therapies | http://dx.doi.org/10.1016/S1360-8592%2803%2900068-8 | Not SYSTEMATIC review |
| Nonsurgical treatment is effective for carpal tunnel syndrome | Anonymous. | 2004 | Journal of Family Practice |  | Not SYSTEMATIC review |
| Hand splints in rehabilitation | Paternostro-Sluga, Tatjana; Stieger, Martina | 2004 | Critical Reviews in Physical and Rehabilitation Medicine | http://dx.doi.org/10.1615/CritRevPhysRehabilMed.v16.i4.10 | Not SYSTEMATIC review |
| Which nonsurgical treatments for carpal tunnel syndrome are beneficial? | Piehl, Janet H. | 2003 | American Family Physician |  | Not SYSTEMATIC review |
| The role of splinting and rehabilitation in the treatment of carpal and cubital tunnel syndromes | Sailer, S.M. | 1996 | Hand Clinics |  | Not SYSTEMATIC review |
| Carpal tunnel syndrome - a review of the literature. | Di Leo G; Vanti C | 2010 | Scienza Riabilitativa |  | Not English |
| Conservative chiropractic approaches to carpal tunnel syndrome. | Barsten G; McCarthy K | 1999 | Topics in Clinical Chiropractic |  | Wrong study design |
| POEMs. Nonsurgical treatment effective for carpal tunnel. |  | 2004 | JAAPA: Journal of the American Academy of Physician Assistants (Haymarket Media, Inc.) |  | No full-text |
| What is the best nonsurgical therapy for carpal tunnel syndrome? | Aukerman, Doug; Lewis, Daniel; Sullo, Elaine | 2010 | Evidence-Based Practice |  | Not SYSTEMATIC review |
| Efficacy of Surgical Vs. Non-Surgical Treatment of Carpal Tunnel Syndrome (Cts): A Systematic Review. | Goyal, R; Kaneria, J; Rai, MK; Bhutani, MK; Singh, R; Rana, P; Rai, M K; Bhutani, M K | 2015 | Value in Health | 10.1016/j.jval.2015.09.2255 | Wrong intervention |
| Efficacy, safety, and cost of surgical versus nonsurgical treatment for carpal tunnel syndrome: A systematic review and meta-analysis: | Retraction...Ren YM, Wang XS, Wei ZJ. Efficacy, safety, and cost of surgical versus nonsurgical treatment for carpal tun | 2017 | Medicine | 10.1097/MD.0000000000006778 | Retracted |
| Ergonomic and physiotherapeutic interventions for treating work-related complaints of the arm, neck or shoulder in adults. A Cochrane systematic review. | Verhagen AP; Karels C; Bierma-Zeinstra SM; Feleus A; Dahaghin S; Burdorf A; De Vet HC; Koes BW | 2007 | Europa Medicophysica |  | Wrong patient population |
| Ergonomic and physiotherapeutic interventions for treating work‚Äêrelated complaints of the arm, neck or shoulder in adults | Verhagen, AP; Karels, CC; Bierma‚ÄêZeinstra, SMA; Burdorf, LL; Feleus, A; Dahaghin, SSD; de Vet, HCW; Koes, BW | 2009 | Cochrane Database of Systematic Reviews | 10.1002/14651858.CD003471.pub4 | Wrong patient population |

**Appendix III- Citation Mapping (Matrix) of the Primary Studies**

| **Primary Studies and modality** | **Included Systematic Reviews** | | | | | | | | | | | | | | | | |
| --- | --- | --- | --- | --- | --- | --- | --- | --- | --- | --- | --- | --- | --- | --- | --- | --- | --- |
|  | Bekhet 2017 (LLLT) | Bula-Oyola 2021 (All EM) | Burger 2017 (LLLT) | Cheung 2020 (LLLT) | Fallah 2017 (LLLT) | Franke 2018 (LLLT) | Fu 2019 (SWD, MWD) | Fulop 2010 (LLLT) | Huisstede 2018 (All EM) | Kim 2019 (ESWT) | Li 2020 (ESWT) | Li 2016 (LLLT) | Page 2013 (US) | Rankin 2017 (LLLT) | Robertson 2001 (US) | Roll 2017 (All EM) | Xie 2022 (ESWT) |
| Abid Ali 2012 (LLLT) | No | Yes | Yes | No | No | No | N/A | No | No | N/A | N/A | No | N/A | No | N/A | No | N/A |
| Aigner 1999 (LLLT) | No | No | No | No | No | No | N/A | No | No | N/A | N/A | No | N/A | Yes | N/A | No | N/A |
| Amirjani 2009 (Iontophoresis) | N/A | No | N/A | N/A | N/A | N/A | N/A | N/A | Yes | N/A | N/A | N/A | N/A | N/A | N/A | No | N/A |
| Arikan 2011 (PMF) | N/A | Yes | N/A | N/A | N/A | N/A | N/A | N/A | Yes | N/A | N/A | N/A | N/A | N/A | N/A | No | N/A |
| Armagan 2014 (US+SP) | N/A | Yes | N/A | N/A | N/A | N/A | N/A | N/A | Yes | N/A | N/A | N/A | No | N/A | No | No | N/A |
| Atthakomol 2018 (ESWT) | N/A | No | N/A | N/A | N/A | N/A | N/A | N/A | No | Yes | Yes | N/A | N/A | N/A | N/A | No | No |
| Atya 2011 (LLLT) | No | Yes | No | No | No | No | N/A | No | No | N/A | N/A | No | N/A | Yes | N/A | No | Yes |
| Badur 2020 (SWD+SP) | N/A | Yes | N/A | N/A | N/A | N/A | No | N/A | No | N/A | N/A | N/A | N/A | N/A | N/A | No | N/A |
| Bakhtiary 2013 (Iontophoresis) | N/A | No | N/A | N/A | N/A | N/A | N/A | N/A | Yes | N/A | N/A | N/A | N/A | N/A | N/A | Yes | N/A |
| Bakhtiary 2004 (US, LLLT) | No | Yes | No | No | No | No | N/A | No | No | N/A | N/A | No | Yes | Yes | No | No | N/A |
| Barbosa 2016 (LLLT, SP) | No | No | Yes | No | No | Yes | N/A | No | No | N/A | N/A | No | N/A | No | N/A | No | N/A |
| Baysal 2006 (US+SP) | N/A | Yes | N/A | N/A | N/A | N/A | N/A | N/A | No | N/A | N/A | N/A | Yes | N/A | No | No | N/A |
| Bilgici 2010 (US) | N/A | No | N/A | N/A | N/A | N/A | N/A | N/A | No | N/A | N/A | N/A | Yes | N/A | No | No | N/A |
| Boyaci 2014 (SWD+SP) | N/A | Yes | N/A | N/A | N/A | N/A | Yes | N/A | Yes | N/A | N/A | N/A | N/A | N/A | N/A | No | N/A |
| Carter 2002 (SMF) | N/A | Yes | N/A | N/A | N/A | N/A | N/A | N/A | No | N/A | N/A | N/A | N/A | N/A | N/A | No | N/A |
| Casale 2013 (LLLT, TENS) | No | Yes | No | No | No | Yes | N/A | No | No | N/A | N/A | No | N/A | Yes | N/A | No | N/A |
| Chang 2019 (ESWT) | N/A | N/A | N/A | N/A | N/A | N/A | N/A | N/A | No | No | No | N/A | N/A | N/A | N/A | No | Yes |
| Chang 2008 (LLLT) | Yes | Yes | Yes | No | Yes | Yes | N/A | No | No | N/A | N/A | Yes | N/A | Yes | N/A | Yes | N/A |
| Chen 2015 (PRF + SP) | N/A | Yes | N/A | N/A | N/A | N/A | N/A | N/A | Yes | N/A | N/A | N/A | N/A | N/A | N/A | No | N/A |
| Colbert 2010 (SMF) | N/A | Yes | N/A | N/A | N/A | N/A | N/A | N/A | Yes | N/A | N/A | N/A | N/A | N/A | N/A | No | N/A |
| Dakowicz 2011 (LLLT, PMF) | No | Yes | No | No | No | Yes | N/A | No | No | N/A | N/A | No | N/A | Yes | N/A | Yes | N/A |
| Dincer 2009 (LLLT, US) | No | Yes | No | Yes | No | No | N/A | No | No | N/A | N/A | No | Yes | Yes | No | No | N/A |
| Duymaz 2012 (US) | N/A | No | N/A | N/A | N/A | N/A | N/A | N/A | No | N/A | N/A | N/A | Yes | N/A | No | No | N/A |
| Ebenbichler 1998 (US) | N/A | No | N/A | N/A | N/A | N/A | N/A | N/A | No | N/A | N/A | N/A | Yes | N/A | Yes | No | N/A |
| Evcik 2007 (LLLT) | Yes | Yes | Yes | Yes | No | Yes | N/A | No | No | N/A | N/A | Yes | N/A | Yes | N/A | No | N/A |
| Ekim 2007 (LLLT) | No | No | No | No | No | No | N/A | Yes | No | N/A | N/A | No | Yes | Yes | No | No | N/A |
| Frasca 2011 (MWD) | N/A | No | N/A | N/A | N/A | N/A | Yes | N/A | Yes | N/A | N/A | N/A | N/A | N/A | N/A | No | N/A |
| Fusakul 2014 (LLLT) | Yes | Yes | Yes | Yes | Yes | Yes | N/A | No | No | N/A | N/A | Yes | N/A | Yes | N/A | No | N/A |
| Gurkay 2012 (Iontophoresis) | N/A | No | N/A | N/A | N/A | N/A | N/A | N/A | Yes | N/A | N/A | N/A | N/A | N/A | N/A | Yes | N/A |
| Incebiyik 2015 (SWD) | N/A | No | N/A | N/A | N/A | N/A | Yes | N/A | No | N/A | N/A | N/A | N/A | N/A | N/A | No | N/A |
| Irvine 2004 (LLLT) | No | No | Yes | No | Yes | Yes | N/A | No | No | N/A | N/A | No | N/A | Yes | N/A | No | N/A |
| Jiang 2011 (LLLT) | No | Yes | Yes | No | Yes | Yes | N/A | No | No | N/A | N/A | No | N/A | Yes | N/A | No | N/A |
| Jothi 2019 (US+SP) | N/A | Yes | N/A | N/A | N/A | N/A | N/A | N/A | No | N/A | N/A | N/A | No | N/A | No | No | N/A |
| Ke 2016 (ESWT) | N/A | Yes | N/A | N/A | N/A | N/A | N/A | N/A | No | Yes | No | N/A | N/A | N/A | N/A | No | Yes |
| Koca 2014 (TENS, IFC) | N/A | Yes | N/A | N/A | N/A | N/A | N/A | N/A | Yes | N/A | N/A | N/A | N/A | N/A | N/A | No | N/A |
| Koyuncu 1995 (US) | N/A | No | N/A | N/A | N/A | N/A | N/A | N/A | No | N/A | N/A | N/A | Yes | N/A | No | No | N/A |
| Lazovic 2014 (LLLT) | Yes | Yes | No | No | Yes | Yes | N/A | No | No | N/A | N/A | No | N/A | Yes | N/A | No | N/A |
| Lucantoni 1992 (LLLT) | No | No | No | No | No | No | N/A | No | No | N/A | N/A | No | N/A | Yes | N/A | No | N/A |
| Michlovitz 2004 (HWT) | N/A | No | N/A | N/A | N/A | N/A | N/A | N/A | Yes | N/A | N/A | N/A | N/A | N/A | N/A | No | N/A |
| Milicin 2018 (US+TT+ES) | N/A | Yes | N/A | N/A | N/A | N/A | N/A | N/A | No | N/A | N/A | N/A | No | N/A | No | No | N/A |
| Notarnicola 2015 (ESWT) | N/A | No | N/A | N/A | N/A | N/A | N/A | N/A | Yes | Yes | No | N/A | N/A | N/A | N/A | No | Yes |
| Oskouei 2014 (TENS+US+SP) | N/A | No | N/A | N/A | N/A | N/A | N/A | N/A | Yes | N/A | N/A | N/A | No | N/A | No | No | N/A |
| Oztas 1998 (US) | N/A | Yes | N/A | N/A | N/A | N/A | N/A | N/A | No | N/A | N/A | N/A | Yes | N/A | No | No | N/A |
| Ozcete 2013 (SWD) | N/A | No | N/A | N/A | N/A | N/A | Yes | N/A | No | N/A | N/A | N/A | N/A | N/A | N/A | No | N/A |
| Paoloni 2015 (ESWT, US) | N/A | Yes | N/A | N/A | N/A | N/A | N/A | N/A | Yes | No | No | N/A | No | N/A | No | No | Yes |
| Piravej 2004 (US) | N/A | No | N/A | N/A | N/A | N/A | N/A | N/A | N/A | No | N/A | N/A | Yes | N/A | No | No | N/A |
| Pratelli 2015 (LLLT) | No | Yes | No | No | No | No | N/A | No | Yes | N/A | N/A | No | N/A | Yes | N/A | No | N/A |
| Raeissadat 2014 (PPNL) | N/A | Yes | N/A | N/A | N/A | N/A | N/A | N/A | Yes | N/A | N/A | N/A | N/A | N/A | N/A | No | N/A |
| Raissi 2017 (ESWT+SP) | N/A | Yes | N/A | N/A | N/A | N/A | N/A | N/A | No | Yes | No | N/A | N/A | N/A | N/A | No | Yes |
| Rayegani 2013 (LLLT) | Yes | No | No | Yes | No | Yes | N/A | No | No | N/A | N/A | Yes | N/A | Yes | N/A | No | N/A |
| Rioja 2012 (LLLT) | No | No | No | No | No | Yes | N/A | No | No | N/A | N/A | No | N/A | No | N/A | No | N/A |
| Rodrigues 2013 (LLLT) | No | No | No | No | No | No | N/A | No | No | N/A | N/A | No | N/A | Yes | N/A | No | N/A |
| Saeed 2012 (LLLT, US) | No | Yes | No | No | No | Yes | N/A | No | No | N/A | N/A | No | No | Yes | No | No | N/A |
| Sawan 2013 (LLLT) | No | No | No | No | No | No | N/A | No | No | N/A | N/A | No | N/A | No | No | Yes | N/A |
| Seok 2013 (ESWT) | N/A | No | N/A | N/A | N/A | N/A | N/A | N/A | Yes | Yes | Yes | N/A | N/A | N/A | N/A | No | Yes |
| Shooshtari 2008 (LLLT) | Yes | Yes | Yes | No | No | Yes | N/A | No | No | N/A | N/A | Yes | N/A | Yes | N/A | No | N/A |
| Soltani 2014 (LLLT) | No | No | No | No | No | Yes | N/A | No | No | N/A | N/A | No | N/A | Yes | N/A | No | N/A |
| Sweilam 2019 (ESWT) | N/A | No | N/A | N/A | N/A | N/A | N/A | N/A | No | No | Yes | N/A | N/A | N/A | N/A | No | No |
| Tao 2018 (ESWT) | N/A | No | N/A | N/A | N/A | N/A | N/A | N/A | No | No | Yes | N/A | N/A | N/A | N/A | No | No |
| Tascioglu 2012 (LLLT) | Yes | Yes | Yes | No | Yes | Yes | N/A | No | No | N/A | N/A | Yes | N/A | Yes | N/A | Yes | N/A |
| Tikiz 2013 (LLLT) | No | No | No | No | No | Yes | N/A | No | No | N/A | N/A | No | N/A | Yes | N/A | No | N/A |
| Vahdatpour 2016 (ESWT) | N/A | No | N/A | N/A | N/A | N/A | N/A | N/A | No | Yes | No | N/A | N/A | N/A | N/A | No | Yes |
| Weintraub 2008 (SMF+PMF) | N/A | Yes | N/A | N/A | N/A | N/A | N/A | N/A | Yes | N/A | N/A | N/A | N/A | N/A | N/A | No | N/A |
| Wolny 2016 (US+LLLT) | No | Yes | No | No | No | No | N/A | No | No | N/A | N/A | No | No | No | No | No | N/A |
| Wu 2016 (ESWT) | N/A | Yes | N/A | N/A | N/A | N/A | N/A | N/A | Yes | No | No | N/A | N/A | N/A | N/A | No | Yes |
| Xu 2020 (ESWT) | N/A | No | N/A | N/A | N/A | N/A | N/A | N/A | No | No | Yes | N/A | N/A | N/A | N/A | No | Yes |
| Yagci 2009 (LLLT) | Yes | No | No | Yes | No | Yes | N/A | No | No | N/A | N/A | Yes | N/A | No | N/A | No | N/A |
| Yildiz 2011 (US) | N/A | No | N/A | N/A | N/A | N/A | N/A | N/A | No | N/A | N/A | N/A | Yes | N/A | No | No | N/A |
| ***List of abbreviations:*** *US, ultrasound; LLLT, low-level laser therapy; SMF, static magnetic field; PMF, pulsed magnetic field; ESWT, extracorporeal shockwave therapy; TT, thermotherapy; ES, electrical stimulation; PRF, pulsed radiofrequency; PPNL, polarized polychromatic noncoherent light therapy; SWD, shortwave diathermy; MWD, microwave diathermy; HWT, heat wrap therapy;* | | | | | | | | | | | | | | | | |  |
